# Genome-wide identification and expression analysis of phytochrome gene family in Aikang58 wheat (*Triticum aestivum* L.)

**DOI:** 10.3389/fpls.2024.1520457

**Published:** 2025-01-21

**Authors:** Zhu Yang, Wenjie Kan, Ziqi Wang, Caiguo Tang, Yuan Cheng, Dacheng Wang, Yameng Gao, Lifang Wu

**Affiliations:** ^1^ Science Island Branch, University of Science and Technology of China, Hefei, Anhui, China; ^2^ The Center for Ion Beam Bioengineering & Green Agriculture, Hefei Institutes of Physical Science, Chinese Academy of Sciences, Hefei, Anhui, China

**Keywords:** wheat, phytochromes, expression profiles, abiotic stresses, co-expression network, LASSO regression

## Abstract

Phytochromes are essential photoreceptors in plants that sense red and far-red light, playing a vital role in regulating plant growth and development through light signal transduction. Despite extensive research on phytochromes in model plants like *Arabidopsis* and rice, they have received relatively little attention in wheat. In this study, we employed bioinformatics methods to identify eight *TaAkPHY* genes in the Aikang58 wheat variety. Based on gene structure, conserved domains, and phylogenetic relationships, the *TaAkPHY* gene family exhibits a high degree of conservation. Synteny analysis revealed the evolutionary history of the *PHY* genes in Aikang58 and Chinese Spring wheat (*Triticum aestivum* L.), barley (*Hordeum vulgare* L.), rice (*Oryza sativa* L.), maize (*Zea mays* L.), quinoa (*Chenopodium quinoa* Willd.), soybean [*Glycine max* (L.) Merr.], and *Arabidopsis* [*Arabidopsis thaliana* (L.) Heynh.]. Among these species, wheat is most closely related to barley, followed by rice and maize. The *cis*-acting element analysis indicates that the promoter regions of *TaAkPHY* genes contain a large number of CAT-box, CGTCA-motif, GC-motif, etc., which are mainly involved in plant development, hormone response, and stress response. Gene expression profiling demonstrated that *TaAkPHY* genes exhibit varying expression levels across different tissues and are induced by various stress conditions and plant hormone treatments. Co-expression network analysis suggested that *TaAkPHY* genes may specifically regulate downstream genes associated with stress responses, chloroplast development, and circadian rhythms. Additionally, the least absolute shrinkage and selection operator (LASSO) regression algorithm in machine learning was used to screen transcription factors such as bHLH, WRKY, and MYB that influenced the expression of *TaAkPHY* genes. This method helps to quickly extract key influencing factors from a large amount of complex data. Overall, these findings provide new insights into the role of phytochromes in wheat growth, development, and stress responses, laying a foundation for future research on phytochromes in wheat.

## Introduction

Phytochromes are a crucial class of plant photoreceptors, which play a key role in regulating plant growth and development in response to changing environmental conditions. Phytochromes function as dimeric proteins, with each monomer comprising an N-terminal photoreceptor domain and a C-terminal dimerization domain (Bae and Choi, 2008; Jones and Quail, 1986). The photoreceptor domain binds covalently to a chromophore, a tetrapyrrole structure composed of four pyrrole rings, enabling the phytochrome with the ability to absorb red and far-red light (Kreslavski et al., 2018). The C-terminal domain primarily mediates dimerization and signal transduction (Jones and Quail, 1986; Edgerton and Jones, 1992). Phytochromes undergo reversible photoconversion between their active state (Pfr) and inactive state (Pr). Upon exposure to red light, the Pr form in the cytoplasm undergoes a structural change, converting into the Pfr form, which then translocates to the nucleus. In its active state, phytochrome can interact with other proteins in the nucleus or cytoplasm to regulate phototransduction pathways (Bae and Choi, 2008; Li et al., 2011). Interestingly, recent studies suggest that phytochromes in the Pr state may also exhibit biological activity within the nucleus (Li and Hiltbrunner, 2021).

Phytochromes are widely present in higher plants, with their apoproteins encoded by a small and divergent gene family. Different types of phytochromes exhibit unique biochemical and physiological characteristics and are involved in various light signaling pathways. For example, *Arabidopsis thaliana* contains five phytochrome-encoding genes, labeled *PHYA* to *PHYE* (Whitelam et al., 1998). The *PHYA* encodes the apoprotein (phyA) of a photolabile phytochrome, which rapidly depletes when exposed to light. The apoproteins (phyB–E) encoded by *PHY* (*B*–*E*) are stable under light conditions and belong to photostable phytochromes (Whitelam et al., 1998). Furthermore, the proteins encoded by these genes each fulfill distinct roles in light sensing and signal transduction, regulating a range of light-dependent responses, including flowering time, shade avoidance, and seed germination (Tripathi et al., 2019; Paik and Huq, 2019). For instance, phyA and phyB regulate flowering time by influencing the transcriptional activation of the flowering induction gene *FLOWERING LOCUS T* (*FT*) (Lei et al., 2024). PhyB is the most important photoreceptor for the shade avoidance response, whereas phyD and phyE also play a certain redundant role; in contrast, phyA is important for shade tolerance (Tripathi et al., 2019; Fraser et al., 2016; Casal, 2013). Under appropriate light conditions, *Arabidopsis* seeds germinate through phyA signaling at very low light fluence, followed by phyD and phyE, whereas seeds less sensitive to light require higher photon fluence to germinate through phyB (Tripathi et al., 2019; Arana et al., 2014; Ibarra et al., 2013). PhyC in *Arabidopsis* requires the assistance of other phytochromes to function (Hu et al., 2013). Likewise, various phytochromes have their own regulatory systems, and the same phytochrome can have different regulatory mechanisms in different plants. For example, monocots, like rice, possess three phytochrome genes, named *PHYA* to *PHYC* (Sun et al., 2017). Among the encoded proteins, phyB and phyC work together to promote flowering, while in *Arabidopsis*, phyB inhibits flowering (Tripathi et al., 2019; Osugi et al., 2011). In wheat, the absence of phyB and phyC prolongs the flowering time (Pearce et al., 2016). Moreover, during the inductive photoperiod of *Arabidopsis* and rice, the loss-of-function mutations in phyA do not affect flowering, whereas in garden pea, flowering is delayed under both short-day and long-day conditions (Tripathi et al., 2019; Woods et al., 2014).

Phytochromes not only play an important regulatory role in growth and development but also participate in various stress responses (Qiu et al., 2023). Under high-temperature stress, phyB acts as both a light receptor and a temperature sensor, promoting plant thermomorphogenesis by regulating the PIF4 (PHYTOCHROME-INTERACTING FACTOR 4) (Bianchetti et al., 2020; Bernardo-García et al., 2014; Foreman et al., 2011). The absence of *PHYB* in *Arabidopsis* enhances thermal tolerance (Qiu et al., 2023; Song et al., 2017).

In drought conditions, phytochromes can enhance the ability of Photosystem I (PSI) and Photosystem II (PSII) to scavenge reactive oxygen species (ROS) through PIFs or promote stomatal closure to reduce transpiration rates and increase abscisic acid (ABA) levels, initiating the expression of drought-related genes, thereby improving drought resistance (Qiu et al., 2023; Li et al., 2020; Wang et al., 2022; Gao et al., 2018b; Huang et al., 2022; Gao et al., 2018a). In rice, the mutant of phytochrome B negatively regulates drought tolerance by modulating stomatal density and total leaf area (Liu et al., 2012). In *Arabidopsis*, phyB increases ABA sensitivity by modulating the expression of *ABCG22*, *PYL5*, *RAB18*, and *RD29A*, facilitating adaptation to drought stress (González et al., 2012).

Under salt stress, phyA/phyB in *Arabidopsis* promotes salt tolerance by enhancing PIF1/PIF3 phosphorylation and degradation (Ma et al., 2023a). The mutants with defects in phyA or phyB in tobacco showed improved salt tolerance compared to wild types, indicating that the *PHYA* and *PHYB* genes in tobacco negatively regulate salt tolerance (Yang et al., 2018). These studies demonstrate that there is a complex interdependent regulatory network among members of the phytochrome family, where signaling pathways integrate and jointly regulate plant growth (Monte et al., 2003).

Weighted Gene Co-expression Network Analysis (WGCNA) is a systems biology approach used to describe the correlation patterns among genes across microarray samples (Langfelder and Horvath, 2008). In a gene co-expression network, genes with similar expression patterns across samples are considered to be part of the same co-expression network or module, with their relationships defined by correlation coefficients (Liu et al., 2021). WGCNA can be employed to identify highly correlated gene clusters (modules), facilitating network-based gene screening methods that can help identify candidate biomarkers (Langfelder and Horvath, 2008).

In omics studies, researchers often face issues of collinearity and high dimensionality due to the large number of sample features; therefore, it is crucial to select independent variables that significantly impact the dependent variable without multicollinearity (Xi et al., 2023). In this context, the least absolute shrinkage and selection operator (LASSO) regression analysis demonstrates unique advantages. By introducing a penalty term, this method effectively shrinks the regression coefficients of unnecessary variables to zero during the model estimation process, thereby eliminating these variables and optimizing variable selection (Xi et al., 2023; Zou and Hastie, 2005). Currently, this method is primarily applied in the medical field, mainly for identifying characteristic biomarkers of diseases (Yang et al., 2024; Cui et al., 2024; Kang et al., 2021).

Although phytochromes have been explored in model plants, there is still insufficient systematic research on the functions, expression regulation, and regulatory networks of the phytochrome gene family in economically important crops such as wheat. The recent publication of the Aikang58 wheat reference genome and annotations offers new data support, helping to enhance our understanding of the role of phytochrome genes in wheat (Jia et al., 2023). This study revealed the distribution and evolution of *PHY* genes in different plants through a genome-wide analysis of Aikang58 and Chinese Spring wheat, along with other plants. Additionally, by utilizing publicly available transcriptome data from Aikang58 wheat, we analyzed the expression patterns of the *TaAkPHY* genes and their regulatory networks under various stress conditions. Finally, we identified potential transcription factors that may regulate the expression of phytochrome genes using machine learning methods. This provides new directions for further research on phytochromes and offers important scientific evidence for the genetic improvement of wheat and enhancing crop adaptability to environmental changes.

## Results

### Identification and phylogenetic analysis of the *PHY* gene family

In this study, we identified a total of eight *PHY* genes in the Aikang58 wheat using two distinct methods, HMMER and BLASTP. These genes were renamed based on their physical positions on the chromosomes and designated as *TaAkPHY1* to *TaAkPHY8* (**Supplementary Figure S1**). Similarly, in Chinese Spring wheat, barley, and maize, eight, three, and six *PHY* genes were identified, named *TaPHY1* to *TaPHY8*, *HvPHY1* to *HvPHY3*, and *ZmPHY1* to *ZmPHY6*, respectively. In soybean and quinoa, eight and three *PHY* genes were identified, named *GmPHY1* to *GmPHY8* and *CqPHY1* to *CqPHY3*, respectively. The *PHY* genes in rice were designated as *OsPHY1* to *OsPHY3*, and those in *Arabidopsis* were named *AtPHY1* to *AtPHY5*. The molecular weights of these PHY proteins ranged from 108.45 kDa to 132.23 kDa, with theoretical isoelectric points between 5.35 and 6.42 (**Supplementary Figure S2**; **Supplementary Table S1**).

**Figure 1:**
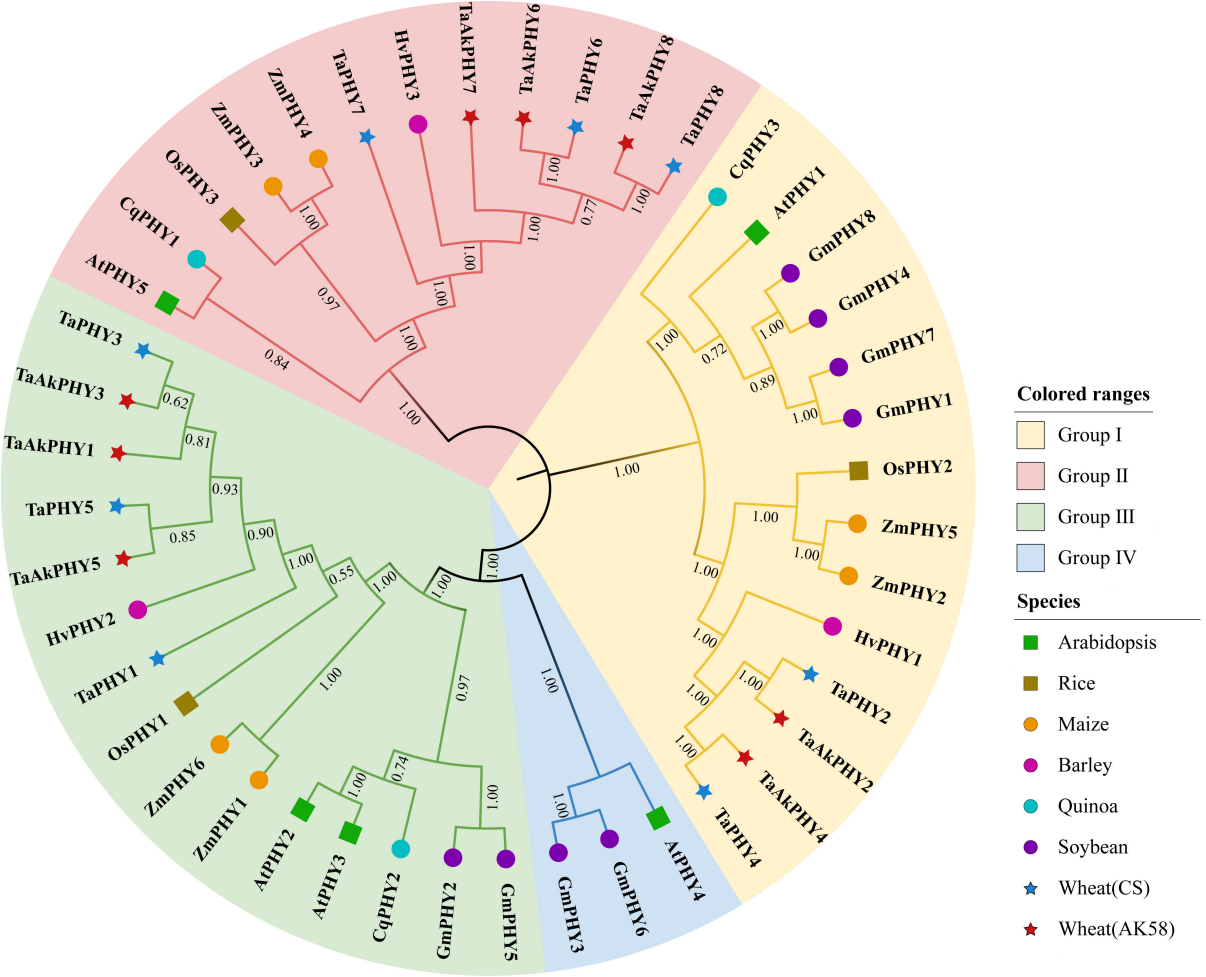
Phylogenetic tree of PHYs. The full-length amino acid sequences of PHYs from Aikang58 and Chinese Spring wheat, *Arabidopsis*, rice, maize, barley, quinoa, and soybean were used for neighbor-joining (NJ) phylogenetic reconstruction with 1,000 bootstrap replicates.

To explore the phylogenetic relationships of the *PHY* gene family among Aikang58 and Chinese Spring wheat and six other plants, a maximum likelihood phylogenetic tree was constructed using MEGA11 based on 44 PHY protein sequences. As shown in **Figure 1**, a total of 44 PHYs were divided into four groups (I, II, III, and IV). Apart from GmPHY3 and GmPHY6 in soybean and AtPHY4 in *Arabidopsis* belonging to group IV, the other plant PHYs are distributed in groups I, II, and III. For Aikang58 wheat, TaAkPHY2 and TaAkPHY4 belong to group I; TaAkPHY6, TaAkPHY7, and TaAkPHY8 belong to group II; and TaAkPHY1, TaAkPHY3, and TaAkPHY5 belong to group III. The grouping of the eight PHYs in Chinese Spring wheat is similar to that of Aikang58 wheat. In addition, the three-dimensional structures of the eight TaAkPHY proteins were predicted using Swiss-Model. The Ramachandran plot showed that most of the points were located within the allowed region, indicating the structural validity of these proteins (**Supplementary Figure S3**). Structural differences were observed among the TaAkPHY proteins in different groups, with the greatest structural variation observed in the TaAkPHYs of group II (TaAkPHY6, 7, and 8), which also supports the rationality of this grouping.

### Structure and distribution of *PHY* genes

**Figure 2:**
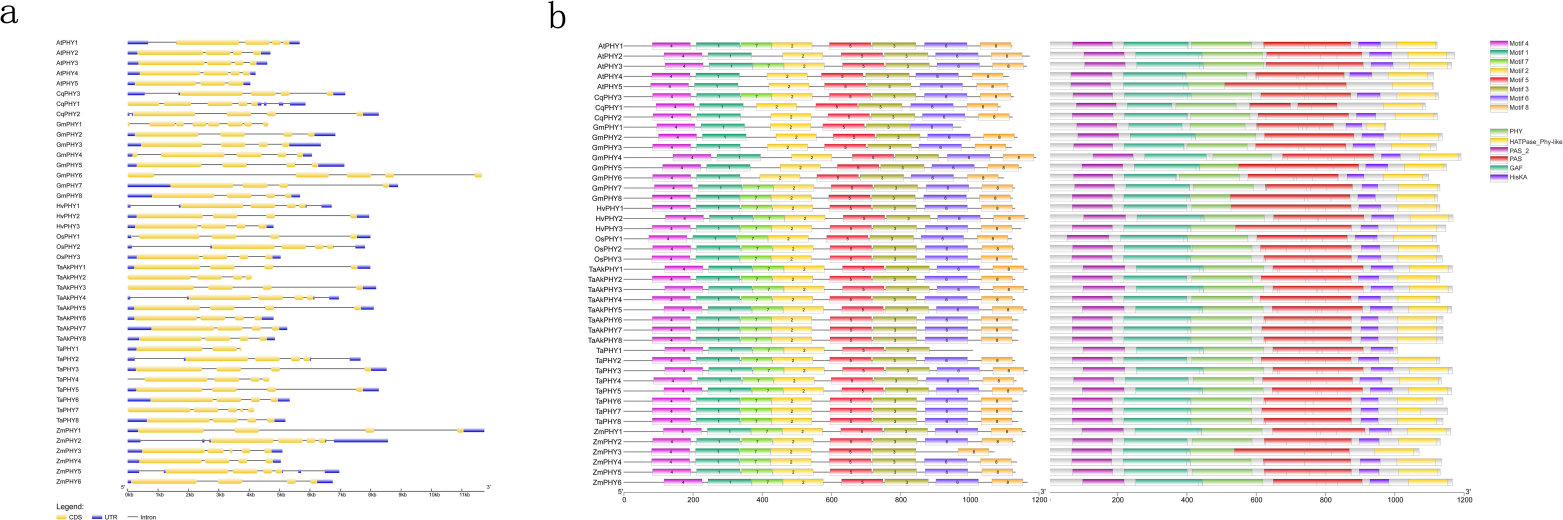
Structure and protein conserved domains of 44 *PHY* genes. **(A)** The structure of 44 *PHY* genes, where yellow boxes represent exons, black lines represent introns, and blue boxes denote untranslated regions (UTRs). **(B)** MEME motifs and conserved domains.

By analyzing the Gene Structure Display Server diagram of the 44 *PHYs*, the number of exons in *PHY* genes varies from three to eight, with 36 *PHY* genes having four exons. The *TaPHY1* and *AtPHY5* genes each have only three exons, while *GmPHY1* has eight exons. *CqPHY1*, *GmPHY4*, *GmPHY6*, *TaPHY7*, and *ZmPHY3* each have five exons (**Figure 2A**). With a view to further exploring the potential functions of PHYs, we used the MEME motif search tool to predict the conserved motifs of PHY proteins from eight plants, identifying a total of eight motifs. Using the Pfam online tool to analyze these motifs revealed that motif 4 is a PAS domain, motifs 1 and 7 are GAF domains, motif 2 is a PHY domain, motifs 3 and 5 are consecutive tandem PAS domains, and motifs 6 and 8 are His kinase domains (**Figure 2B**). Previous studies have shown that the N-terminal of phytochrome proteins serves as the light-sensing module, containing PAS, phytochrome-specific PHY, and GAF domains. The C-terminal functions as the output module, comprising two tandem PAS domains (PRD and PAS repeat domain) and a histidine kinase-related domain (HKRD) (Cheng et al., 2021). The identified PHY proteins are consistent with the known conserved domains of phytochrome proteins.

### Synteny analysis among *TaAkPHY* genes

**Figure 3:**
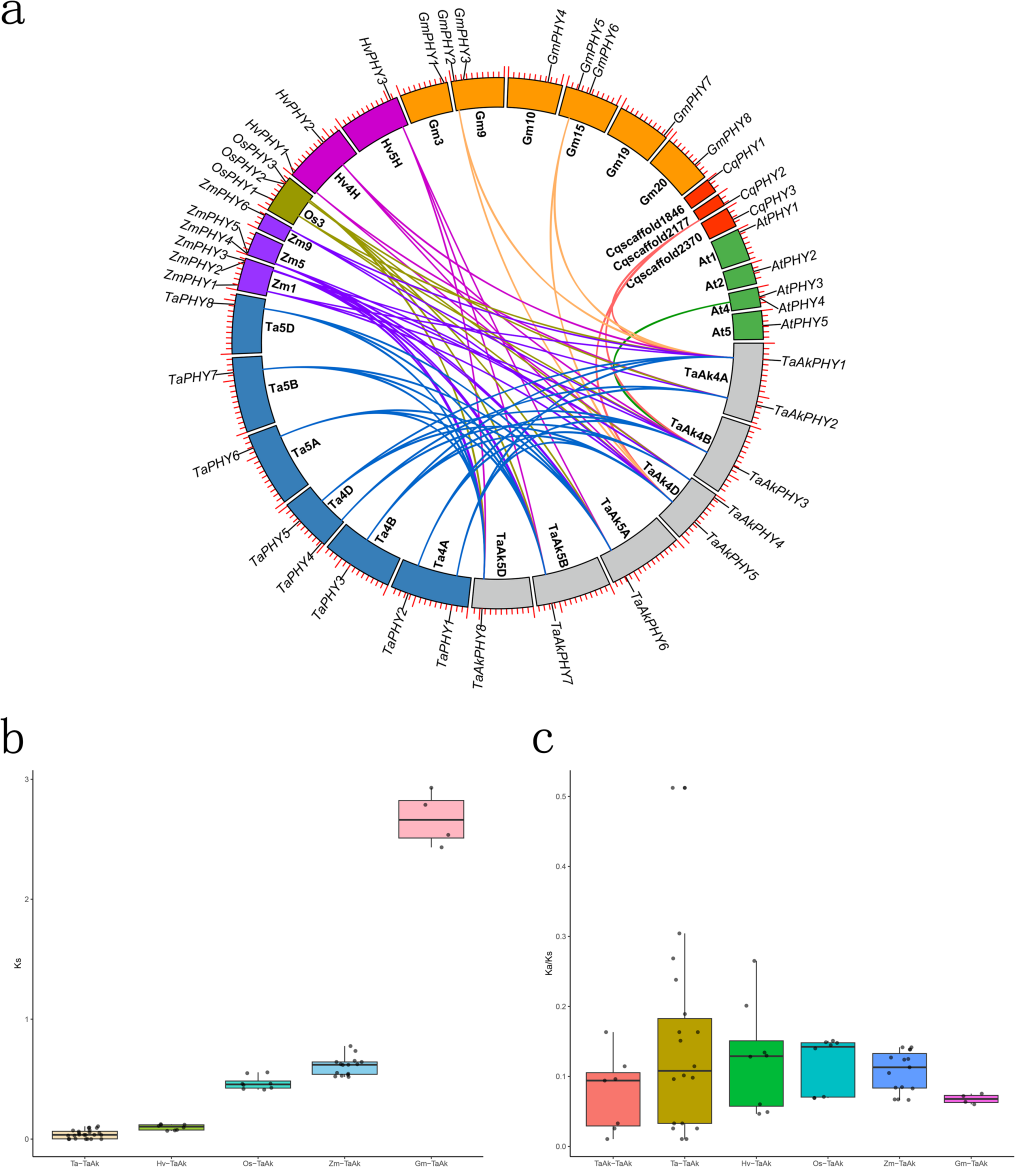
Collinearity analysis comparing Aikang58 wheat with Chinese Spring wheat and six other plants. **(A)** Blue represents Chinese Spring wheat, purple represents maize, yellow-brown represents rice, dark purple represents barley, orange represents soybean, red represents quinoa, green represents *Arabidopsis*, and gray represents Aikang58 wheat. **(B)** Ks values of homologous pairs among different species. **(C)** Ka/Ks values of homologous pairs.

To gain insight into the evolutionary relationships of *PHY* genes among Aikang58 wheat, Chinese Spring wheat, barley, rice, maize, soybean, quinoa, and *Arabidopsis*, we conducted a genome synteny analysis. The results identified 24 pairs of orthologous genes between Aikang58 wheat and Chinese Spring wheat, eight pairs with barley, eight pairs with rice, 15 pairs with maize, four pairs with soybean, three pairs with quinoa, and one pair with *Arabidopsis* (**Figure 3A**; **Supplementary Table S2**). The average Ks values between Aikang58 wheat and the orthologous pairs of Chinese Spring wheat, barley, rice, maize, and soybean were 0.043, 0.098, 0.467, 0.609, and 2.67, respectively (**Figure 3B**; **Supplementary Table S2**). Aikang58 wheat has accumulated significant sequence divergence between the orthologous pairs with *Arabidopsis* and quinoa, including both synonymous and non-synonymous mutations, which makes it hard to calculate the Ks values (**Supplementary Table S2**). These results indicate that *TaAkPHY*s are the most highly conserved in the genomes of Aikang58 wheat and Chinese Spring wheat, followed by barley, while quinoa and *A. thaliana* show the least conservation. To investigate the selective pressure on *PHY* genes, the Ka/Ks values of the orthologous gene pairs were calculated. All Ka/Ks ratios of the homologous gene pairs (both orthologous and paralogous) within Aikang58 wheat among Chinese Spring wheat, barley, rice, maize, and soybean were less than 1 (**Figure 3C**; **Supplementary Table S2**), indicating that these *PHY* genes have undergone negative selection in these plants. This suggests that *PHY* genes are crucial for plant survival and adaptation, requiring stable maintenance of their functions.

### Identification of *cis*-acting element in promoter region of the *TaAkPHY* genes

**Figure 4:**
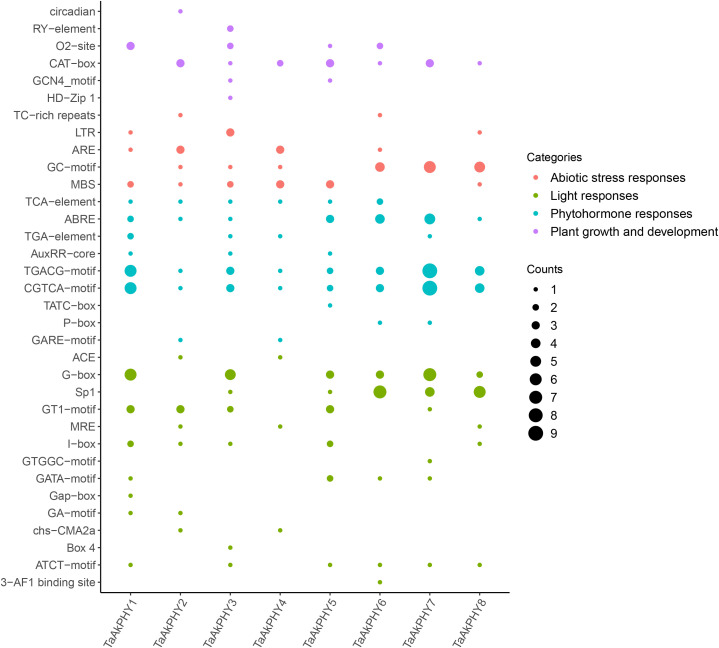
*cis*-Elements in the promoter region of the *TaAkPHY* genes. The size of the dots represents the number of elements, and the color indicates different response types.

To analyze the *cis*-regulatory elements in the 2,000-bp upstream region of the transcription start site of the *TaAkPHY* genes, we identified a series of *cis*-acting elements related to plant hormone responses, plant growth and development, light responses, and abiotic stress responses (**Figure 4**). The *cis*-acting elements related to hormone response include abscisic acid response elements (ABREs) and methyl jasmonate (MeJA) response elements (CGTCA-motif). The elements that respond to ABA are mainly found in the promoter regions of *TaAkPHY6* and *TaAkPHY7*, while those that respond to MeJA are primarily distributed in *TaAkPHY1* and *TaAkPHY7* (**Figure 4**; **Supplementary Table S3**). In the *cis*-regulatory elements related to growth and development, the meristem expression-related regulatory element (CAT-box) is the most abundant in promoter region, along with other elements such as the endosperm expression regulatory element (O2-site) and the palisade mesophyll cell differentiation regulatory element. The O2-site elements are most abundant in group III members (*TaAkPHY1*, *TaAkPHY3*, and *TaAkPHY5*), while CAT-box elements are distributed in groups I and II (*TaAkPHY2*, *TaAkPHY4*, *TaAkPHY6*, *TaAkPHY7*, and *TaAkPHY8*) (**Figure 4**; **Supplementary Table S3**). Light-responsive elements include G-box, GT1-motif, SP1, Box 4, GATA-motif, and I-box. The light-responsive elements are mainly distributed in the *TaAkPHY*s of groups II and III, with the highest number of G-box elements totaling 26 (**Figure 4**; **Supplementary Table S3**). Furthermore, we identified various *cis*-regulatory elements related to abiotic stress responses in the *TaAkPHY* promoter region, including drought-induced elements (MBS), low temperature-related elements (LTR), defense- and stress-related elements (TC-rich repeats), and hypoxia-specific induced elements (GC-motif and ARE). Notably, the numbers of MBS and GC-motif elements are relatively high, at 12 and 18, respectively (**Figure 4**; **Supplementary Table S3**). Among these eight *TaAkPHY* genes, the *TaAkPHY6*, *7*, and *8* promoter regions contain the highest number of abiotic stress-responsive *cis*-regulatory elements, suggesting that these three *TaAkPHY* genes may play a regulatory role in plant stress responses (**Supplementary Table S3**).

### SSR and targeted miRNAs of the *TaAkPHY* genes

Among the eight *TaAkPHY* genes identified, simple sequence repeats (SSRs) were detected only in *TaAkPHY1*, *TaAkPHY3*, *TaAkPHY4*, and *TaAkPHY7*. Specifically, *TaAkPHY1*, *TaAkPHY3*, and *TaAkPHY7* each contained one trinucleotide-type SSR, while *TaAkPHY4* contained two mononucleotide-type SSRs (**Supplementary Table S4**). Subsequently, we used the psRNATarget server to predict the potential miRNA targets of these eight *TaAkPHY* genes. The results showed that the *TaAkPHY* genes in groups I, II, and III were regulated by different miRNAs. For example, tae-miR9666b-3p, tae-miR9657a-3p, and tae-miR1137a targeted the *TaAkPHY* genes in group II (*TaAkPHY6*, *7*, and *8*); tae-miR9774, tae-miR5048-5p, and tae-miR5049-3p targeted the *TaAkPHY* genes in group I (*TaAkPHY2* and *TaAkPHY4*); tae-miR9679-5p, tae-miR1122c-3p, and tae-miR1128 targeted the *TaAkPHY* genes in group III (*TaAkPHY1*, *3*, and *5*). Additionally, tae-miR9673-5p and tae-miR9657b-5p were able to target *TaAkPHY* genes from different subgroups. Notably, tae-miR9674b-5p specifically targeted *TaAkPHY2*, tae-miR1127b-3p and tae-miR9668-5p targeted *TaAkPHY3*, tae-miR9661-5p and tae-miR171a targeted *TaAkPHY4*, tae-miR6197-5p and tae-miR1121 primarily targeted *TaAkPHY5*, and tae-miR9672b exclusively targeted *TaAkPHY6* (**Supplementary Figure S4**; **Supplementary Table S5**).

### Expression pattern of transcriptome analysis

**Figure 5:**
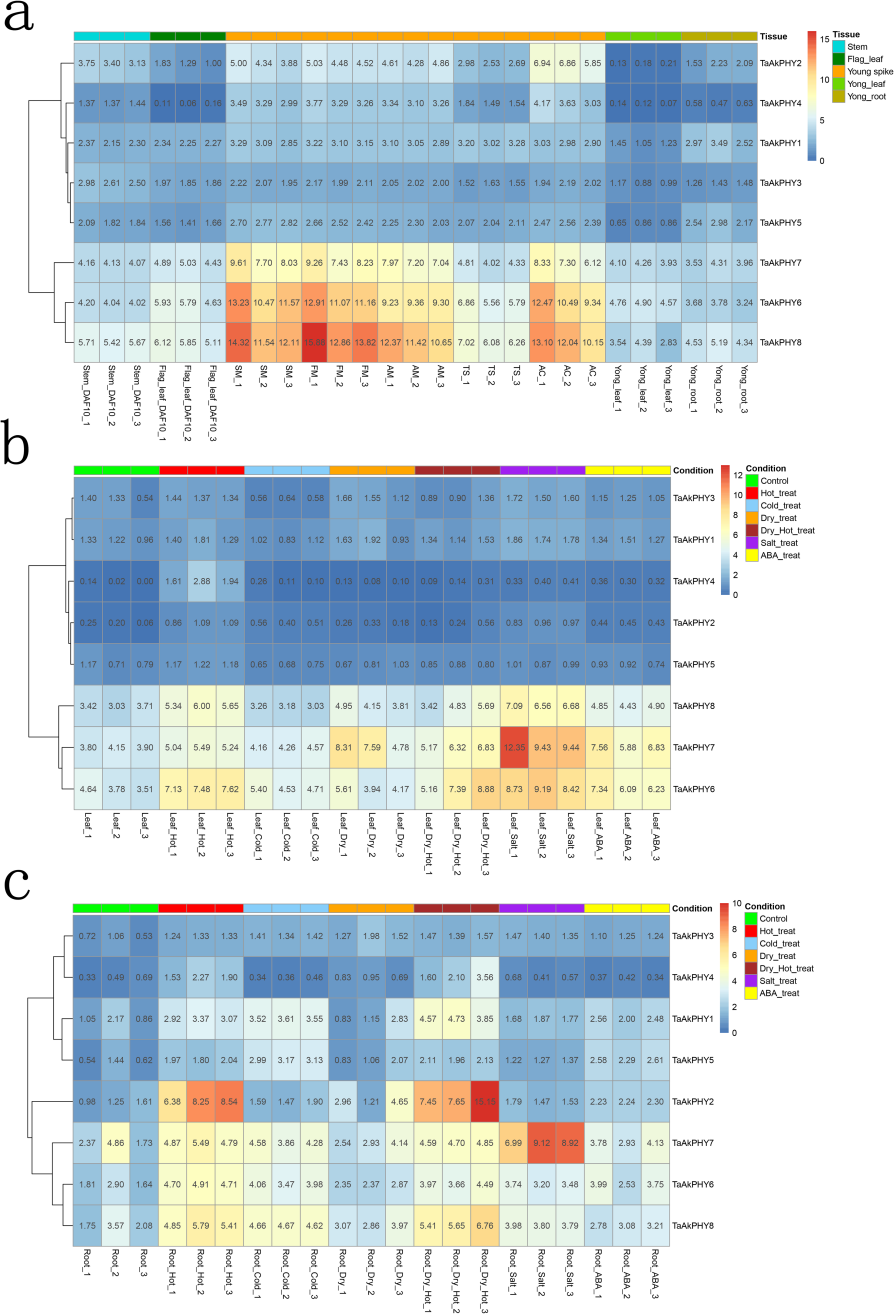
Heatmap of *TaAkPHY* gene expression profiles. Red symbolizes high expression level, and blue represents low expression level. **(A)** Expression levels of *TaAkPHY*s in different tissues. The young spikes include five stages: spikelet meristem stage (SM), floret meristem stage (FM), anther primordial stage (AM), tetrad stage (TS), and anther connective stage (AC). **(B)** Expression levels of *TaAkPHY*s in leaves under different stress conditions and abscisic acid (ABA) treatment. **(C)** Expression levels of *TaAkPHY*s in roots under different stress conditions and ABA treatment.

For the purpose of exploring the role of *TaAkPHY*s in wheat development, we analyzed publicly available transcriptome data to investigate their expression patterns in different tissues of Aikang58 wheat, including flag leaves, stems, young spikes, roots, and young leaves. As shown in **Figure 5A**, there are differences in the expression levels of *TaAkPHY*s within the same tissue; specifically, *TaAkPHY6*, *TaAkPHY7*, and *TaAkPHY8* from group II have higher expression levels compared to *TaAkPHY1* to *TaAkPHY5*. In different tissues, members of groups I and II (*TaAkPHY2*, *TaAkPHY4*, *TaAkPHY6*, *TaAkPHY7*, and *TaAkPHY8*) exhibit higher expression levels in spikes, while members of group III (*TaAkPHY1*, *TaAkPHY3*, and *TaAkPHY5*) show similar expression levels in leaves, stems, spikes, and roots.

Recent studies have highlighted the crucial role of the phytochrome signaling pathway in plant responses to abiotic stresses such as high temperature, low temperature, drought, and salinity (Qiu et al., 2023). ABA is an inhibitory hormone that also plays a key regulatory role in plant responses to both biotic and abiotic stresses (Raghavendra et al., 2010). Furthermore, *cis*-acting element analysis of *TaAkPHY*s promoters indicates the presence of multiple stress-responsive and ABA-responsive elements. Therefore, we analyzed the responses of *TaAkPHY*s to abiotic stresses and ABA treatments using publicly available RNA-seq data (a total of 42 samples, with experimental conditions including leaves and roots subjected to heat, cold, drought, salt stress, and ABA treatments). The results show that in leaves, under drought, cold, heat, salt stress, and ABA treatment conditions, the expression levels of group II members (*TaAkPHY6*, *TaAkPHY7*, and *TaAkPHY8*) were significantly increased in five treatments compared to the control. In contrast, the expression levels of *TaAkPHY1* to *TaAkPHY5* did not change significantly (**Figure 5B**). In root tissues, the expression trends of *TaAkPHY6*, *TaAkPHY7*, and *TaAkPHY8* under the same experimental treatment conditions were similar to those in leaves, suggesting that they may play important roles in responding to these stresses and regulating the stress response mechanism. However, under heat stress conditions, *TaAkPHY2* showed a significant increase in expression levels in root tissues, while its expression change in leaves was not significant, indicating that *TaAkPHY2* may have a specific stress response mechanism in different tissues (**Figure 5C**).

### Analysis of weighted gene co-expression network

With the aim of understanding the regulatory network of *TaAkPHY*s under stress conditions, RNA-seq data from root and leaf tissues of the Aikang58 wheat variety (a total of 83 samples) were used under drought, heat, cold, salt, and ABA treatment conditions, and a co-expression network was constructed using WGCNA. After removing genes with low expression [Transcripts Per Million (TPM) < 1], a total of 36,486 genes were used to build a scale-free co-expression network with a soft threshold power of β = 10 (**Supplementary Figure S5A**). Using the dynamic tree cut method, 20 modules were identified. *TaAkPHY1*, *TaAkPHY2*, and *TaAkPHY5* belong to the turquoise module; *TaAkPHY4* and *TaAkPHY8* belong to the red module; *TaAkPHY3*, *TaAkPHY6*, and *TaAkPHY7* belong to the pink module, brown module, and blue module, respectively; the turquoise, pink, red, brown, and blue modules contain 9,799, 1,149, 2,492, 3,201, and 496 genes, respectively (**Supplementary Figure S5B**). To further investigate the biological processes and metabolic pathways of the genes in the modules, Gene Ontology (GO) and Kyoto Encyclopedia of Genes and Genomes (KEGG) enrichment analyses were performed for the modules containing *TaAkPHY*s. The genes in these modules are primarily involved in biological processes such as intracellular transport, transcription regulation, ion transport, and photosynthesis, as well as pathways including mRNA surveillance, carotenoid biosynthesis, the Calvin cycle, and circadian rhythms (**Supplementary Figures S6**, **S7**; **Supplementary Tables S6**, **S7**).

**Figure 6:**
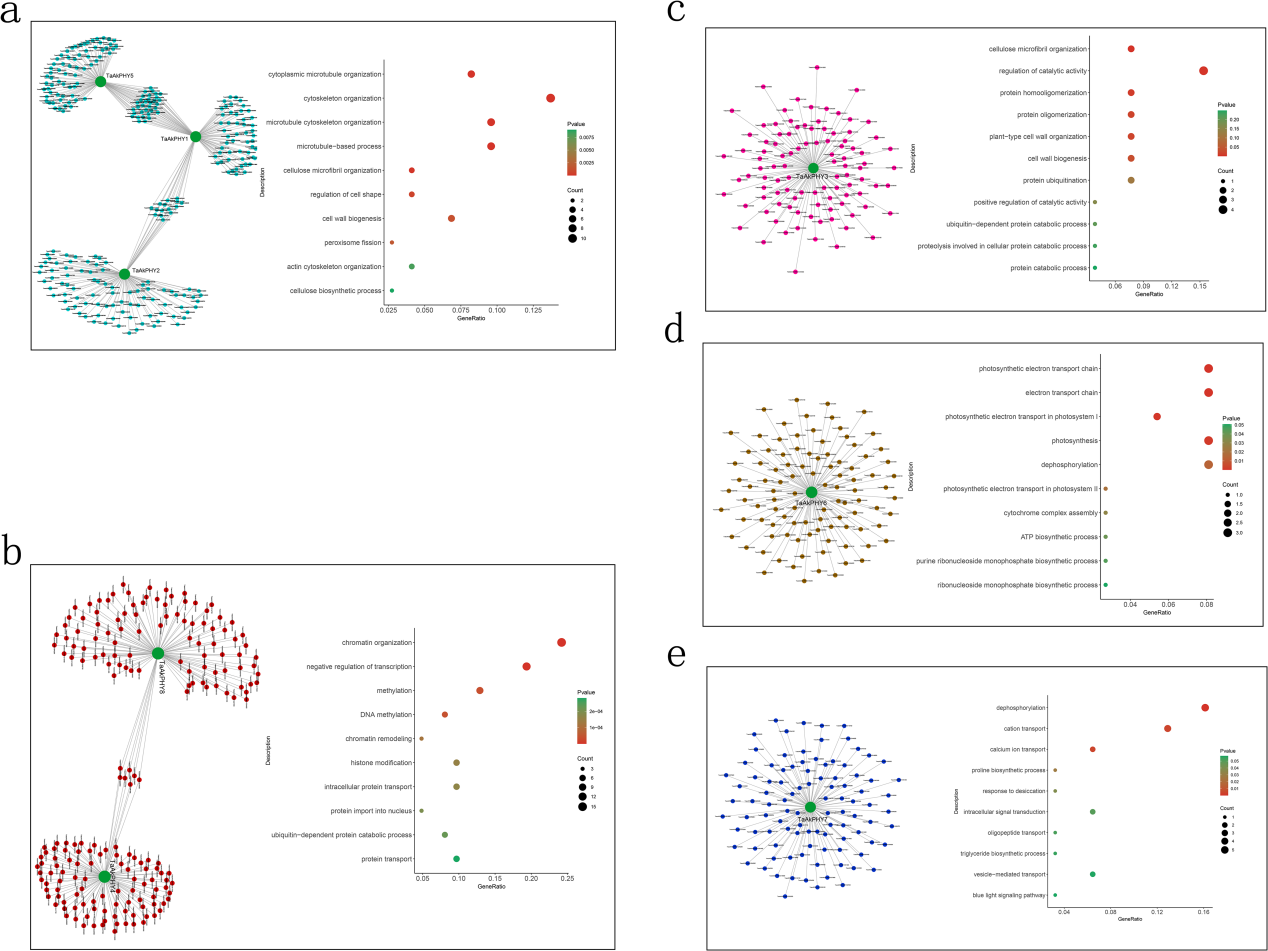
The co-expression network of the *TaAkPHY* gene and Gene Ontology (GO) annotation analysis. Through Weighted Gene Co-expression Network Analysis (WGCNA), the top 100 co-expressed genes related to *TaAkPHY1*, *2*, and *5* [turquoise module; **(A)**], *TaAkPHY4* and *8* [red module; **(B)**], *TaAkPHY 3* [pink module; **(C)**], *TaAkPHY 6* [brown module; **(D)**] were selected based on topological overlap matrix (TOM) values from their respective modules. These genes were then subjected to GO annotation analysis.

In order to visualize the co-expression network of the eight *TaAkPHY*s, we selected the top 100 genes with the highest topological overlap matrix (TOM) values of correlation with *TaAkPHY*s in the modules and constructed a gene regulatory network using the Cytoscape software. Subsequently, GO analysis was conducted on the genes highly associated with *TaAkPHY*s (**Figure 6**). The results showed that the genes related to *TaAkPHY1*, *TaAkPHY2*, and *TaAkPHY5* are predominantly involved in biological processes such as microtubule and cytoskeleton organization, regulation of cell shape, and cell wall synthesis. Genes related to these processes include *TraesAK58CH5A01G454400*, *TraesAK58CH3B01G191200*, and *TraesAK58CH4A01G130000* (**Figure 6A**; **Supplementary Table S8**). Additionally, the genes associated with *TaAkPHY2* and *TaAkPHY4* are primarily involved in biological processes such as chromatin remodeling, histone modification, and transcriptional regulation. Among these, *TraesAK58CH7A01G204200* and *TraesAK58CH2D01G153500* are related to chromatin remodeling, while *TraesAK58CH5B01G104100* and *TraesAK58CH3B01G089800* are linked to histone modification and transcriptional regulation (**Figure 6B**; **Supplementary Table S8**). The genes related to *TaAkPHY3* are primarily enriched in processes related to protein oligomerization and cellulose microfibril organization, such as *TraesAK58CH4A01G092200*, *TraesAK58CH4D01G243200*, *TraesAK58CH6D01G478800*, and *TraesAK58CH6A01G386000* (**Figure 6C**; **Supplementary Table S8**). The genes associated with *TaAkPHY6* primarily participate in biological processes such as photosynthesis and the electron transport chain. The involved genes include *TraesAK58CH5B01G575700*, *TraesAK58CH2D01G264000*, and *TraesAK58CH5D01G628100* (**Figure 6D**; **Supplementary Table S8**). Finally, the genes highly correlated with *TaAkPHY7* are mainly associated with processes involving the transport of calcium ions and other cations, as well as drought stress responses. Specifically, *TraesAK58CH5B01G116500*, *TraesAK58CH3B01G316500*, *TraesAK58CH4A01G166900*, and *TraesAK58CH5A01G109000* are involved in ion transport, while *TraesAK58CH5A01G217500* plays a role in drought stress response (**Figure 6E**; **Supplementary Table S8**).

Additionally, we identified the top five genes with the highest TOM values for each of the eight *TaAkPHY*s to explore the functions of these highly correlated genes. We used the Basic Local Alignment Search Tool (BLAST) search tool to find homologous genes in Chinese Spring wheat, rice, and *Arabidopsis*; since many genes in rice and wheat have not been studied extensively, we inferred their potential functions based on reports from *Arabidopsis* (**Supplementary Table S9**). The results indicate that genes significantly correlated with *TaAkPHY*s include *TraesAK58CH5A01G454400* (WVD2/WDL family protein) and *TraesAK58CH7B01G002800* (COP1-interacting protein-like protein), both of which may play a role in light-induced hypocotyl elongation. Additionally, *TraesAK58CH4A01G497000* (PEX11 gene family) and *TraesAK58CH7A01G581800* (encodes a peroxisomal catalase) are potentially linked to peroxisomes. Genes *TraesAK58CH2D01G599800* (synaptotagmin family protein) and *TraesAK58CH6B01G174400* (calcium-dependent lipid-binding family protein) are associated with calcium ion signal response, while *TraesAK58CH4A01G450000* [encodes a bifunctional protein that has 3′(2′),5′-bisphosphate nucleotidase and inositol polyphosphate 1-phosphatase activities], *TraesAK58CH6A01G141500* (encodes ClpB4), and *TraesAK58CH3B01G342900* (encoded protein is highly correlated with heat stress-related proteins HSP17.4B, HSP101, and DNAJ) are related to stress responses. *TraesAK58CH2D01G241800*, *TraesAK58CH2A01G216200*, and *TraesAK58CH2B01G240900* are all homologous genes to *Thylakoid formation1* (*Thf1*) in *Arabidopsis* (**Supplementary Tables S8**, **S9**).

### Screening hub transcription factor influencing *TaAkPHY* expression through machine learning

**Figure 7:**
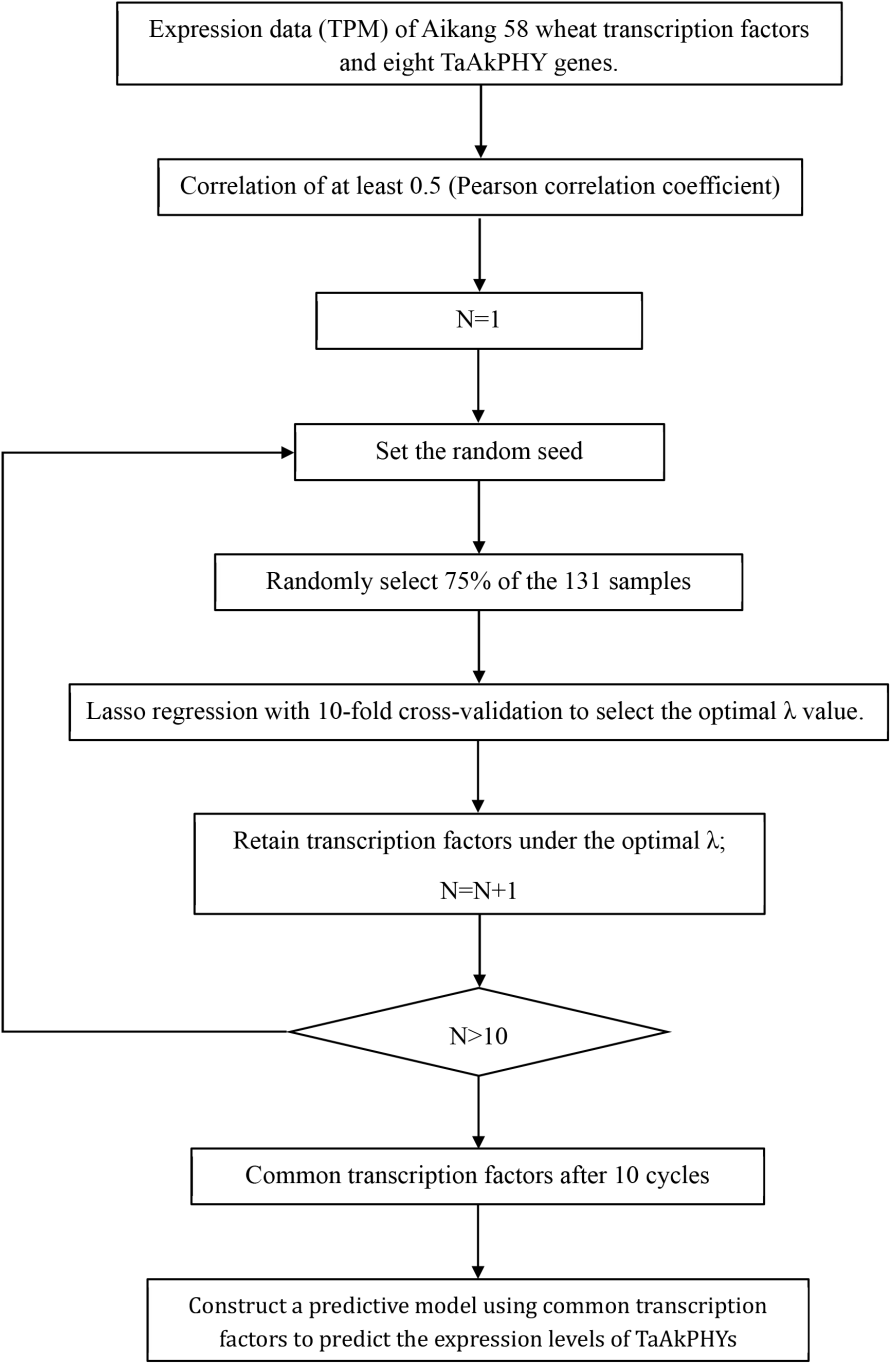
Screening process for transcription factors that affect the expression of *TaAkPHY* genes.

Transcriptional regulation forms the basis for shaping plant growth, development, and environmental adaptability. Transcription factors, which are DNA-binding proteins, are key elements in transcriptional regulation (Strader et al., 2022). They control chromatin and transcription by recognizing specific DNA sequences, creating a complex system that guides genomic expression (Lambert et al., 2018). The activity of transcription factors determines cellular functions and responses to environmental changes (Vaquerizas et al., 2009). Screening for transcription factors helps to unveil gene regulation mechanisms, understand how plants adapt to environmental changes, and advance gene function research. While existing studies mostly focus on phytochrome as core elements in light signal transduction, which regulate downstream gene expression by interacting with various factors, there has been less attention on the regulation of phytochrome gene expression levels itself. Therefore, this study employs LASSO regression machine learning algorithms to identify transcription factors that regulate the expression of *TaAkPHY*s based on samples from 131 samples, including different tissues and various stress treatments (**Figure 7**).

**Figure 8:**
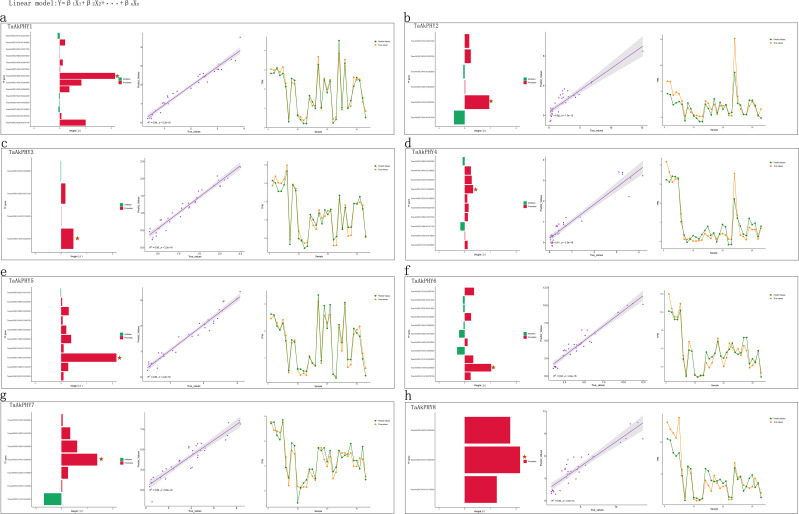
Weights of transcription factors and model evaluation. The bar chart illustrates the regulatory effects of various transcription factors on *TaAkPHY* expression, with the length of the bars representing the magnitude of the β values in the linear model. In the chart, red bars indicate transcription factors that promote *TaAkPHY* expression, while green bars indicate those that inhibit it, with pentagons marking the transcription factor with the highest weight. The scatter plot displays the distribution of predicted versus actual values for the test set, where a higher R^2^ value indicates greater predictive accuracy. **(A–G)** correspond to the analysis results for *TaAkPHY1* to *8*, collectively reflecting the predictive effectiveness of the model established through key transcription factors in regulating *TaAkPHY* expression.

We identified 14, 6, 4, 10, 10, 11, 7, and 3 hub transcription factors in *TaAkPHY1* to *TaAkPHY8*, respectively (**Figure 8**, **Supplementary Table S10**). Subsequently, we constructed predictive models for the expression of each *TaAkPHY* using these transcription factors and evaluated the accuracy of the models using a test set. The R^2^ values of the models were 0.96, 0.81, 0.95, 0.91, 0.96, 0.93, 0.88, and 0.85, respectively (**Figure 8**). This indicates that these transcription factors can serve as important influencing features and effectively predict the expression levels of the *TaAkPHY* genes. According to the coefficients from the linear model, we found that among the 14 transcription factors that significantly affect *TaAkPHY1*, nine factors promote the expression of *TaAkPHY1*, while five factors inhibit its expression (**Figure 8A**; **Supplementary Table S10**). Among these, *TraesAK58CH4B01G347400* has the greatest impact on *TaAkPHY1* and belongs to the bHLH family (**Figure 8A**; **Supplementary Table S11**). Similarly, the analysis of *TaAkPHY2* to *TaAkPHY8* reveals that the genes of the transcription factors with the most significant effects are *TraesAK58CH2A01G200300* (WRKY family), *TraesAK58CH1D01G352700* (MYB family), *TraesAK58CH5D01G289000* (C2H2 family), *TraesAK58CH4B01G347400* (C2C2 family), *TraesAK58CH1B01G324800* (FAR1 family), and *TraesAK58CH4A01G390400* (CAMTA family) (**Figures 8B–H**; **Supplementary Tables S10**, **S11**).

## Discussion

Phytochromes are crucial regulatory proteins that significantly influence plant growth, development, and responses to environmental stresses. In this study, we identified 44 *PHY* genes through whole-genome analysis in Aikang58 and Chinese Spring wheat, as well as barley, rice, maize, quinoa, soybean, and *Arabidopsis*. In the model organism *Arabidopsis*, there are five phytochrome genes: *PHYA*, *PHYB*, *PHYC*, *PHYD*, and *PHYE* (Whitelam et al., 1998). In this study, these correspond to *AtPHY1*, *AtPHY2*, *AtPHY5*, *AtPHY3*, and *AtPHY4*, respectively. Specifically, *AtPHY1*, *AtPHY5*, and *AtPHY4* are classified into groups I, II, and IV, corresponding to *PHYA*, *C*, and *E*, while *AtPHY2* and *AtPHY3*, which are closely related, fall into group III, corresponding to *PHYB/D*. Through phylogenetic tree analysis, we divided these 44 PHYs into four groups (**Figure 1**). Based on the nomenclature of phytochromes in *Arabidopsis*, we classified the PHYs in groups I, II, III, and IV into the subfamilies PHYA, PHYC, PHYB/D, and PHYE. In both monocots and dicots, phytochromes are distributed among the PHYA, PHYC, and PHYB/D subfamilies; however, not all dicotyledonous plants possess the PHYE subfamily, as exemplified by quinoa. This indicates that the phytochromes in the model plant *Arabidopsis* may not fully represent other dicotyledonous plants as well as monocots due to the fact that phytochrome genes may have undergone significant expansions after the divergence of monocots and dicots and experienced multiple independent evolutionary events in dicots (Qiao et al., 2019; Mathews and Sharrock, 1996).

The distribution results of *PHY* genes on chromosomes indicate that *PHY* genes are unevenly distributed across chromosomes or scaffolds in different plants. Wheat is a complex hexaploid plant containing three subgenomes—A, B, and D—with a total of 21 chromosomes (Pont et al., 2013). Aikang58 and Chinese Spring wheat both have eight *PHY* gene members located on chromosomes 4A, 4B, 4D, 5A, 5B, and 5D. In the two wheat varieties, chromosomes 4A and 4D each contain two *PHY* genes, while chromosome 4B has only one *PHY* gene identified. This is because the proteins encoded by the genes *TraesAK58CH4B01G057100* and *TraesCS4B02G052000* on chromosome 4B lack the HKRD that is characteristic of the C-terminal region of phytochromes. Additionally, one *PHY* gene was identified on each of the 5A, 5B, and 5D chromosomes (**Supplementary Figure S1**). *TaAkPHY*s and *TaPHY*s show consistency in their chromosomal distribution and phylogenetic relationships (**Figure 1**; **Supplementary Figure S1**), indicating that the genomes of different wheat varieties may be relatively conserved, sharing a common ancestor, and have undergone gene recombination, mutations, and modifications of redundant genes during the evolutionary process (Levy and Feldman, 2022; Otto, 2007; Chen, 2007).

In gene structure analysis, it was found that *TaPHY1* and *TaPHY7* have three and five exons, respectively, while both *TaAkPHY1* and *TaAkPHY7* have four exons, with *TaAkPHY1* having longer introns (**Figure 2A**). These structural differences in genes may be influenced by transposable elements, which significantly impact genome structure and gene function during plant evolution. Transposable elements reorganize the genome and alter gene structure and regulation through activities such as transposition, insertion, deletion, chromosome breakage, and ectopic recombination (Bennetzen, 2000; Bennetzen and Wang, 2014; Vicient and Casacuberta, 2017). Additionally, the proportion of transposable elements in the wheat genome is very high, accounting for 86%, 85%, and 83% of the sequences in the A, B, and D subgenomes, respectively (Wicker et al., 2018). In tetraploid wild emmer wheat (genome AABB), the insertion sites of miniature inverted-repeat transposable elements (MITEs) vary among different varieties, and this variation has a significant impact on gene structure and expression (Domb et al., 2017). It is noteworthy that the gene structures of most *PHY* genes are highly conserved among different species.

In this study, we further elucidated the evolutionary history of the *PHY* gene family by systematically analyzing the homologous relationships of *PHY* genes between Aikang58 wheat and various monocots (Chinese Spring wheat, barley, rice, and maize) as well as dicots (quinoa, *A. thaliana*, and soybean). Among these, *TaAkPHY1*, *3*, and *5* exhibit homologous pairs with *PHY* genes in quinoa, *Arabidopsis*, and soybean (**Figure 3A**; **Supplementary Table S2**). The average Ks value between Aikang58 wheat and soybean homologous pairs is 2.67 (**Figure 3B**; **Supplementary Table S2**), while the sequence divergence between homologous pairs with quinoa and *Arabidopsis* is too great to calculate the Ks value. The average Ks values of homologous pairs are 0.043, 0.098, 0.467, and 0.609, respectively (**Figure 3B**; **Supplementary Table S2**), indicating that the relationship between Aikang58 wheat and Chinese Spring wheat is the closest, followed by barley, rice, and maize, which corresponds with the divergence times of the species (Ma et al., 2023b).

During the analysis of the *cis*-regulatory elements of the *TaAkPHY* genes, we found that the promoter region of *TaAkPHY*s contains the highest number of light-responsive and hormone-responsive elements (**Supplementary Table S3**). This indicates that the expression abundance of *TaAkPHY*s may be significantly influenced by light and hormones. Previous studies have reported that the transcript abundance of *PHY* in *Arabidopsis* and field-grown grapevine leaves exhibits a diurnal rhythm (Tóth et al., 2001; Kühn et al., 2009). For example, under long-day conditions, the transcript abundance of *VvPHYA* and *VvPHYB* in grapevine leaves fluctuates with the day–night cycle, peaking before dawn and dropping to its lowest level after dusk (Kühn et al., 2009). However, research on the effects of hormones on *PHY* gene expression remains relatively limited.

Subsequently, we explored the expression patterns of *TaAkPHY*s in different tissues and under various stress conditions using transcriptome data. While *TaAkPHY*s are expressed in various tissues of wheat, their expression levels are particularly elevated in the spikes, especially among the members of groups I and II (**Figure 5A**). Research indicates that under long-day (LD) conditions, the heading time of wheat spikes occurs earlier than under short-day (SD) conditions. This acceleration is due to phyB and phyC regulating the transcriptional activation of PPD1 by sensing light signals, thereby speeding up the heading time of wheat (Pearce et al., 2016; Alvarez et al., 2023; Chen et al., 2014; Kippes et al., 2020). This implies that the members of *TaAkPHY*s from groups I and II play an important role in the development of spikes.

Studies have shown that in *Arabidopsis*, *PHYB* modulates freezing tolerance in response to changes in light quality (Pearce et al., 2016; Franklin and Whitelam, 2007). Furthermore, research has found that the phyB-null mutant in wheat exhibits a significant upregulation of cold-regulated (COR) genes (Pearce et al., 2016). These studies indicate that phytochromes are involved in the regulation of stress responses. Under stress conditions, the transcription levels of *TaAkPHY*s showed varying changes, particularly under heat and cold stress, where the expression of *TaAkPHY6*, *TaAkPHY7*, and *TaAkPHY8* was the most significant. However, when leaves and roots were subjected to the same stress, the transcription levels of *TaAkPHY1* to *TaAkPHY5* in the leaves remained almost unchanged, while the levels in the roots showed significant variation (**Figures 5B, C**). This indicates that the sensitivity of phytochrome gene expression to stress response varies in wheat, and different tissues may respond differently to stress. Additionally, under ABA treatment, we found that the transcription levels of *TaAkPHY6* and *TaAkPHY7* significantly increased in both leaves and roots, likely due to a higher number of *cis*-elements responsive to abscisic acid (ABRE) in the promoter regions of these two genes (**Figure 5C**; **Supplementary Table S3**). Therefore, the levels of *TaAkPHY*s transcripts are not only influenced by light but also significantly affected by hormones and stress.

In the WGCNA co-expression network analysis, eight *TaAkPHY* genes were grouped into different modules, with *TaAkPHY1*, *TaAkPHY2*, and *TaAkPHY5* belonging to the same module, while *TaAkPHY4* and *TaAkPHY8* were assigned to another module. It has been reported that phytochromes co-regulate the expression of target genes through different mechanisms, including the formation of homodimers or heterodimers to regulate downstream targets, such as PHYB–PHYB and PHYB–PHYC (Pearce et al., 2016; Klose et al., 2015; Legris et al., 2019). Since genes in the same module are related to each other in terms of expression patterns, *TaAkPHY*s in the same module may be more inclined to form dimers that specifically regulate downstream genes.

Subsequently, we conducted GO enrichment analysis on the genes in the module and those highly associated with *TaAkPHY*s. We found that in the brown module, highly correlated genes with *TaAkPHY6* are primarily related to photosynthesis, suggesting that *TaAkPHY6* is involved in the regulation of photosynthesis (**Figure 6D**; **Supplementary Figure S6D**). Studies have reported that plant photosynthesis can be affected by drought and salt stress conditions, possibly due to changes in the protein activity of photosystems PSII and PSI, or because of stomatal closure that impacts carbon dioxide absorption. This effect leads to a severe imbalance between light capture and energy utilization, significantly affecting the photosynthetic process (Dalal, 2021; Silveira and Carvalho, 2016). This indicates that under stress conditions, *TaAkPHY6* and the genes within its module may enhance the plant’s adaptability to stress by optimizing photosynthesis and energy metabolism, thereby playing a significant role in stress resistance mechanisms.

In addition, the genes in the blue module, including those highly associated with *TaAkPHY7*, mainly participate in ion transport as well as processes related to drought stress (**Figure 6E**; **Supplementary Figure S6E**). Ion transport not only regulates the osmotic pressure of plant cells but also plays a crucial role in how plants respond to stress, particularly in the transport of calcium ions (Ca^2+^) (Schroeder and Hedrich, 1989; Tong et al., 2021). Among the genes highly correlated with *TaAkPHY7*, *TraesAK58CH3B01G316500* and *TraesAK58CH4A01G166900* are involved in the transport of calcium ions (**Supplementary Table S8**). Calcium ions not only are essential nutrients for plants but also serve as important second messengers within cells. Additionally, Ca^2+^ can regulate the homeostasis of potassium ions (K^+^), sodium ions (Na^+^), magnesium ions (Mg^2+^), and iron ions (Fe^3+^) (Wang et al., 2023). Abiotic stress can lead to dynamic changes in intracellular calcium ion concentrations over time and space. Through interactions with calcium-binding proteins, signals are transmitted downstream, activating the expression of related genes and ion transport (Tong et al., 2021; Zhao et al., 2021). Therefore, the genes in this module may primarily enhance the plant’s adaptability to stresses like drought and salinity by regulating intracellular calcium ion concentrations, which in turn affects the concentrations of other ions and maintains cellular stability.

In the process of screening co-expressed genes of *TaAKPHY*s, we found that among the top five highly correlated genes of *TaAkPHY6*, three genes (*TraesAK58CH2D01G241800*, *TraesAK58CH2A01G216200*, and *TraesAK58CH2B01G240900*) correspond to the same homologous gene in *Arabidopsis*, which encodes the Thf1 protein (**Supplementary Table S9**). This protein not only regulates photosynthesis but also is crucial for chloroplast development, particularly in its role in thylakoid stacking. Furthermore, the transcription level of *Thf1* increases under light conditions and decreases in darkness, which is consistent with the expression pattern of phytochrome transcripts (Tóth et al., 2001; Kühn et al., 2009; Wang et al., 2004; Zhan et al., 2016). This suggests that *TaAkPHY6* may greatly impact chloroplast development and photosynthesis in wheat by regulating the transcription of *TraesAK58CH2D01G241800*, *TraesAK58CH2A01G216200*, and *TraesAK58CH2B01G240900*.

Research has found that the transcription levels of phytochrome genes are influenced not only by light exposure (Tóth et al., 2001) but also by hormone and stress treatments (**Figures 5B, C**). Since transcription factors are essential proteins in transcription regulation, studies have shown that the zinc finger domain-containing transcription factor TZP can regulate the abundance of PHYB protein and its transcripts (Fang et al., 2022). Therefore, from the perspective of transcription factors, we screened for transcription factors that affect the transcription levels of *TaAkPHY*s using machine learning methods based on data from 131 samples. This approach not only identifies transcription factors that significantly impact gene transcription but also assesses the influence weight of each transcription factor. The results show that among the 14 transcription factors affecting the transcription level of *TaAkPHY1*, according to annotations from eggNOG-mapper, these genes primarily encode proteins with zinc finger and bHLH structures (**Supplementary Table S11**). Among them, the gene encoding the bHLH transcription factor *TraesAK58CH4B01G347400* has the greatest influence weight on the transcription of *TaAkPHY1* (**Figure 8A**; **Supplementary Table S10**). Research indicates that bHLH transcription factors can specifically bind to G-box elements in the promoter region, thereby regulating gene transcription levels (Qian et al., 2006; Ahmad et al., 2015). Additionally, we found a larger quantity of G-box elements in the promoter region of *TaAkPHY1* and *TaAkPHY7* (**Supplementary Table S3**). The gene *TraesAK58CH4A01G390400*, which significantly affects the expression level of *TaAkPHY7* (**Figure 8G**, **Supplementary Table S10**), encodes a CAMTA transcription factor that can bind to G-box *cis*-elements and further regulate gene expression (Noman et al., 2021). This indicates that *TraesAK58CH4B01G347400* and *TraesAK58CH4A01G390400* may be upstream regulatory genes of *TaAkPHY1* and *TaAkPHY7*, and these findings further validate the reliability of the approach.

For the remaining six transcription factors that significantly affect *TaAkPHY* expression levels, they belong to the WRKY, MYB, zinc finger proteins (C2H2 and C2C2), FAR1, and CAMTA families (**Figures 8B–F, H**; **Supplementary Table S10**). These transcription factors play important regulatory roles in plant growth and development, stress response, and light signal transduction (Noman et al., 2021; Zhang et al., 2023; Xie et al., 2020; Wu et al., 2022, 2017; Liu et al., 2022; Jiang et al., 2017). In addition to these significant transcription factors affecting the expression of *TaAkPHY*s, we also identified other types of transcription factors, such as auxin response factors (ARFs), homeodomain-leucine zipper (HD-ZIP), AP2/ERF, and TCP, which also influence the transcription of *TaAkPHY*s (**Supplementary Tables S10**, **S11**).

Therefore, it is feasible to select transcription factors that significantly affect the expression of *TaAkPHY*s through the LASSO regression algorithm in machine learning. Additionally, the predictive model performed excellently on the test set (**Figure 8**), highlighting the key role of these transcription factors in regulating *TaAkPHY* gene expression and confirming the effectiveness of this method.

## Conclusion

This study identified eight *PHY* genes in Aikang58 wheat using HMMER and BLAST methods and compared them with *PHY* genes from other monocot and dicot plants, revealing the conservation of *PHY* genes across different species. Phylogenetic and synteny analyses demonstrated the evolutionary relationships of *PHY* genes between monocots and dicots. Furthermore, weighted correlation network analysis revealed the regulatory network of *TaAkPHY* genes under stress conditions. GO and KEGG analyses suggest that *TaAkPHY* genes may respond to environmental stress by regulating gene transcription, protein degradation, histone modification, photosynthesis, and ion transport. Additionally, potential transcription factors that may regulate or be regulated by *TaAkPHY* gene expression were identified using the LASSO regression, providing important clues for future exploration of the regulatory mechanisms of *TaAkPHY* expression. In summary, this research enhances understanding of the wheat *PHY* genes family and provides new perspectives on its roles in plant growth, development, and responses to stress. These findings have significant consequences for developing wheat improvement strategies and enhancing the crop’s adaptability to environmental stresses.

## Methods

### Identification of *PHY* gene family members in Aikang58 and Chinese Spring wheat and other four plants

In order to identify the *PHY* gene family, the reference genome information and annotations for Aikang58 wheat were downloaded from Wheatdb (https://triticeae.henau.edu.cn/aikang58/) (Jia et al., 2023). The reference genome information of Chinese Spring wheat (genome assembly: IWGSC, iwgsc_refseqv1.0), barley (genome assembly: MorexV3_pseudomolecules_assembly), rice (genome assembly: IRGSP-1.0), maize (genome assembly: Zm-B73-REFERENCE-NAM-5.0), quinoa (genome assembly: ASM168347v1), soybean (genome assembly: Glycine_max_v2.1), and *Arabidopsis* (genome assembly: TAIR10) were obtained from EnsemblPlants (https://plants.ensembl.org/index.html). To extract protein sequences from these plants, the gffread (v0.12.7) software was used (Pertea and Pertea, 2020). Two methods were used to identify the *PHY* genes in Aikang58 wheat and other five plants. First, the hidden Markov model (HMM) profile of the PHY domain (PF00360) downloaded from Pfam (https://www.ebi.ac.uk/interpro/entry/pfam/) was used to identify PHYs in Aikang58 and Chinese Spring wheat, barley, maize, quinoa, and soybean, and the cutoff E-value was 1e−10 (Finn et al., 2014). Second, all published PHY protein sequences from *Arabidopsis* and rice were regarded as queries to search for TaAkPHYs, TaPHYs, HvPHYs, ZmPHYs, CqPHYs, and GmPHYs by performing BLAST, and the cutoff E-value was 1e−5 (Yin et al., 2024). The protein sequences selected through two methods were further analyzed for their domains using the online websites Pfam and Batch CD-Search (https://www.ncbi.nlm.nih.gov/Structure/), and the candidate PHY proteins were ultimately identified based on the specific domains of phytochromes (Cheng et al., 2021). The physicochemical properties of the PHY protein were calculated and visualized using the online tool Multiple Protein Profiler (MPP) 1.0 (https://mproteinprofiler.microbiologyandimmunology.dal.ca/) (Sganzerla Martinez et al., 2024).

### Phylogenetic analysis, three-dimensional structure prediction, conserved motifs, and gene structure analysis

The ClustalW program was used to align PHY protein sequences (Thompson et al., 1994), and the phylogenetic tree was built using the MEGA 11 program with the neighbor-joining method and 1,000 replicate iterations (Tamura et al., 2021). The three-dimensional structure of the protein was predicted using the Swiss-Model online tool (https://swissmodel.expasy.org/) (Waterhouse et al., 2018). The conserved motifs of PHY proteins were identified using the MEME program (https://meme-suite.org/meme/tools/meme) (Bailey et al., 2009). Domains were identified using Pfam programs (Finn et al., 2014). The exon–intron analysis of the PHY genes was conducted according to the genome annotation file, and a map of the PHY gene structure was generated using the GSDS website (https://gsds.gao-lab.org/) (Hu et al., 2015).

### Chromosomal distribution and synteny analysis

To illustrate the chromosomal distribution of the *PHY* genes in Aikang58, Chinese Spring wheat, and six other plants, a chromosome location map was generated based on the physical positions of the PHY genes on the chromosomes or scaffolds using the Gene Structure View tool (https://github.com/CJ-Chen/TBtools-II/releases) (Chen et al., 2023). Additionally, the MCScanX software was used to identify syntenic pairs among species, and the Advanced Circos software (https://github.com/CJ-Chen/TBtools-II/releases) was utilized to generate synteny plots (Chen et al., 2023; Wang et al., 2012). The DnaSP v5.10.1 software was used to estimate the Ka and Ks of gene duplication pairs and deduce the selective pressure and divergence time for PHY genes in eight plants using the Ka and Ks values (Librado and Rozas, 2009). Generally, the ratio of Ka/Ks greater than 1 indicates the positive selection, equal to 1 means the neutral selection, and less than 1 represents the negative selection (Cannon et al., 2004).

### Promoter *cis*-acting element analysis

We extracted the 2,000-bp sequences upstream of the coding regions of eight *TaAkPHY* genes from their genome. These sequences were then analyzed using PlantCARE (https://bioinformatics.p-sb.ugent.be/webtools/plantcare/html/) to predict putative *cis*-regulatory elements (Lescot, 2002).

### Prediction of SSR and miRNAs targeting *TaAkPHY* genes

The psRNATarget server was used with default parameters to predict potential miRNA targets in the *TaAKPHY* genes (Dai et al., 2018). Interaction networks between miRNAs and *TaAKPHY* genes were drawn using the Cytoscape software (version 3.10.1) (Shannon et al., 2003). SSRs in the *TaAKPHY* genes were obtained from the online MISA-web (https://webblast.ipk-gatersleben.de/misa/) (Beier et al., 2017), with search parameters set as follows: mononucleotides ≥ 10, dinucleotides ≥ 6, trinucleotides ≥ 5, tetranucleotides ≥ 5, pentanucleotides ≥ 5, and hexanucleotides ≥ 5 (Zhang et al., 2022).

### Expression pattern of transcriptome analysis

To gain a deeper understanding of the expression patterns of the *TaAkPHY*s gene in different tissues and under abiotic stress conditions in Aikang58 wheat, RNA-seq data (CRA013077) were obtained from the China National Center for Bioinformation (https://www.cncb.ac.cn/) (Jia et al., 2023). These datasets encompass data from wheat seedling roots and young leaves subjected to drought, cold, salt, and ABA stress treatments, as well as data from different stages of spikes development, flag leaves, stems, and grains. The raw sequence data were trimmed and qualified using Trimmomatic (version 0.39) with default parameters (Bolger et al., 2014). Sequences were aligned to the Aikang58 wheat reference genome (https://triticeae.henau.edu.cn/aikang58/) using hisat2 (version 3.1.0) (D. Kim et al., 2019). Quantification of transcript expression and calculation of TPM values were performed using the featureCounts tool within the Subread (version 2.0.6) software (Liao et al., 2014).

### Analysis of weighted gene co-expression network

In this study, we utilized the WGCNA package in R (v4.3.2) to construct a gene co-expression network for Aikang58 wheat under abiotic stress conditions. Initially, we preprocessed the sample data by filtering out genes with low expression levels. Subsequently, employing the WGCNA package, we constructed a correlation matrix and determined the optimal soft threshold to convert this matrix into an adjacency matrix. Using the adjacency matrix, we generated a TOM. We then grouped genes exhibiting similar expression patterns into modules using hierarchical clustering (core parameters: MEDissThres = 0.25, minimum Module Size = 30) (Langfelder and Horvath, 2008). Additionally, we employed eggNOG-mapper (http://eggnog-mapper.embl.de/) to annotate the protein sequences of Aikang58 wheat genes (Cantalapiedra et al., 2021). Subsequently, we performed GO and KEGG functional enrichment analyses using the “clusterProfiler” R package (v4.3.2) (Yu et al., 2012). These analyses evaluated gene-related biological processes (BPs), molecular functions (MFs), cellular components (CCs), and signaling pathways associated with the genes. We visualized the results of these enrichments using the “ggplot2” R package. Furthermore, we utilized the Cytoscape (v3.10.2) (https://cytoscape.org/) software to visualize the gene co-expression network (Shannon et al., 2003).

### Screening of hub transcription factors that influence the expression of *TaAkPHY*s

We downloaded transcription factor data for Aikang58 wheat from Wheatdb (https://triticeae.henau.edu.cn/aikang58/) (Jia et al., 2023) and utilized the expression levels of transcription factor genes and *TaAkPHY*s from 131 samples in the publicly available RNA-seq dataset (CRA013077) to screen for hub transcription factors using LASSO regression. First, we selected transcription factors with a Pearson’s correlation coefficient of at least 0.5 with the target *TaAkPHY* genes. Subsequently, we used the “glmnet” R package to perform LASSO regression analysis for further screening of these transcription factors (Friedman et al., 2010). The specific method is as follows: we randomly selected 75% of the sample data for LASSO regression, and we employed 10-fold cross-validation to choose the Lambda value with the smallest mean squared error, retaining transcription factors associated with this Lambda value. To ensure result reliability, we repeated this process 10 times with different random seeds, ensuring randomness in each selection. Transcription factors that were consistently retained across all 10 iterations were considered candidate transcription factors. Next, we constructed a linear model using the selected candidate transcription factors as variables to predict the target gene’s expression level, employing 75% of the data for training and 25% for testing, which helped assess the influence of these transcription factors on *TaAkPHY* expression and determine their weights. The linear model is as follows: $Y=\beta_{0}+\beta_{1}x_{1}+$ … $\beta_{n}x_{n}$ , where β represents the weight of hub transcription factors, x represents transcription factor expression levels, and Y represents *TaAkPHY* gene expression levels.

## Data Availability

The datasets presented in this study can be found in online repositories. The names of the repository/repositories and accession number(s) can be found in the article/**Supplementary Material**.

## Supplementary material

Supplementary Figure 1 Chromosomal distribution of PHY genes in Aikang58 **(A)** and Chinese spring wheat **(B)**, as well as in rice **(C)**, maize **(D)**, barley **(E)**, quinoa **(F)**, soybean **(G)**, and Arabidopsis **(H)**.

Supplementary Figure 2 Physicochemical properties of 44 PHYs proteins. These include amino acid length, molecular weight, theoretical isoelectric point, aliphatic index, hydropathicity (GRAVY), aromaticity, charge of pH 7, instability index and stability.

Supplementary Figure 3 3D structural model and Ramachandran plot of the TaAkPHY proteins.

Supplementary Figure 4 Interaction network of targeted miRNAs and *TaAkPHYs*.

Supplementary Figure 5
**(A)** Analysis of network topology for soft threshold power. **(B)** Hierarchical cluster tree showing co-expression modules identified by weighted gene correlation network analysis (WGCNA)

Supplementary Figure 6 Biological processes of GO enrichment for genes in the turquoise **(B)**, pink **(C)**, red **(D)**, brown **(E)**, and blue **(F)** modules.

Supplementary Figure 7 KEGG pathway enrichment of genes in the turquoise **(B)**, pink **(C)**, red **(D)**, brown **(E)**, and blue **(F)** modules.

Supplementary Table 1 Detailed information on 44 PHY proteins.

Supplementary Table 2 Information on the PHY homologous relationships within Aikang58 wheat and between Aikang58 wheat and Chinese Spring wheat, barley, rice, maize, soybean, quinoa, and Arabidopsis.

Supplementary Table 3 Types and numbers of cis-acting elements in the upstream region of the *TaAkPHY* genes in Aikang58 wheat.

Supplementary Table 4 Predicted gene specific SSR markers in the *TaAkPHY* genes.

Supplementary Table 5 Predicted miRNA targets in the *TaAkPHY* genes.

Supplementary Table 6 Gene GO enrichment in turquoise, pink, red, brown, and blue modules.

Supplementary Table 7 Gene KEGG pathway enrichment in turquoise, pink, red, brown, and blue modules.

Supplementary Table 8 GO enrichment of genes co-expressed with the eight *TaAkPHYs*.

Supplementary Table 9 The top five genes associated with each *TaAkPHY* and their homologous genes in Chinese spring wheat, rice, and Arabidopsis.

Supplementary Table 10 Information on the weight of transcription factors affecting the expression of *TaAkPHYs*.

Supplementary Table 11 eggNOG-mapper annotation information for the protein sequences of Aikang 58 wheat genes.

Supplementary Table 12 The *TaAkPHY* genes and their reported orthologous genes in Arabidopsis and rice.
