# Supplementary figures and images for: Genome-wide identification and expression analysis of phytochrome gene family in Aikang58 wheat (Triticum aestivum L.)
Source: Front Plant Sci. 2025 Jan 21;15:1520457. doi: 10.3389/fpls.2024.1520457 (PMC11790602; doi:10.3389/fpls.2024.1520457)

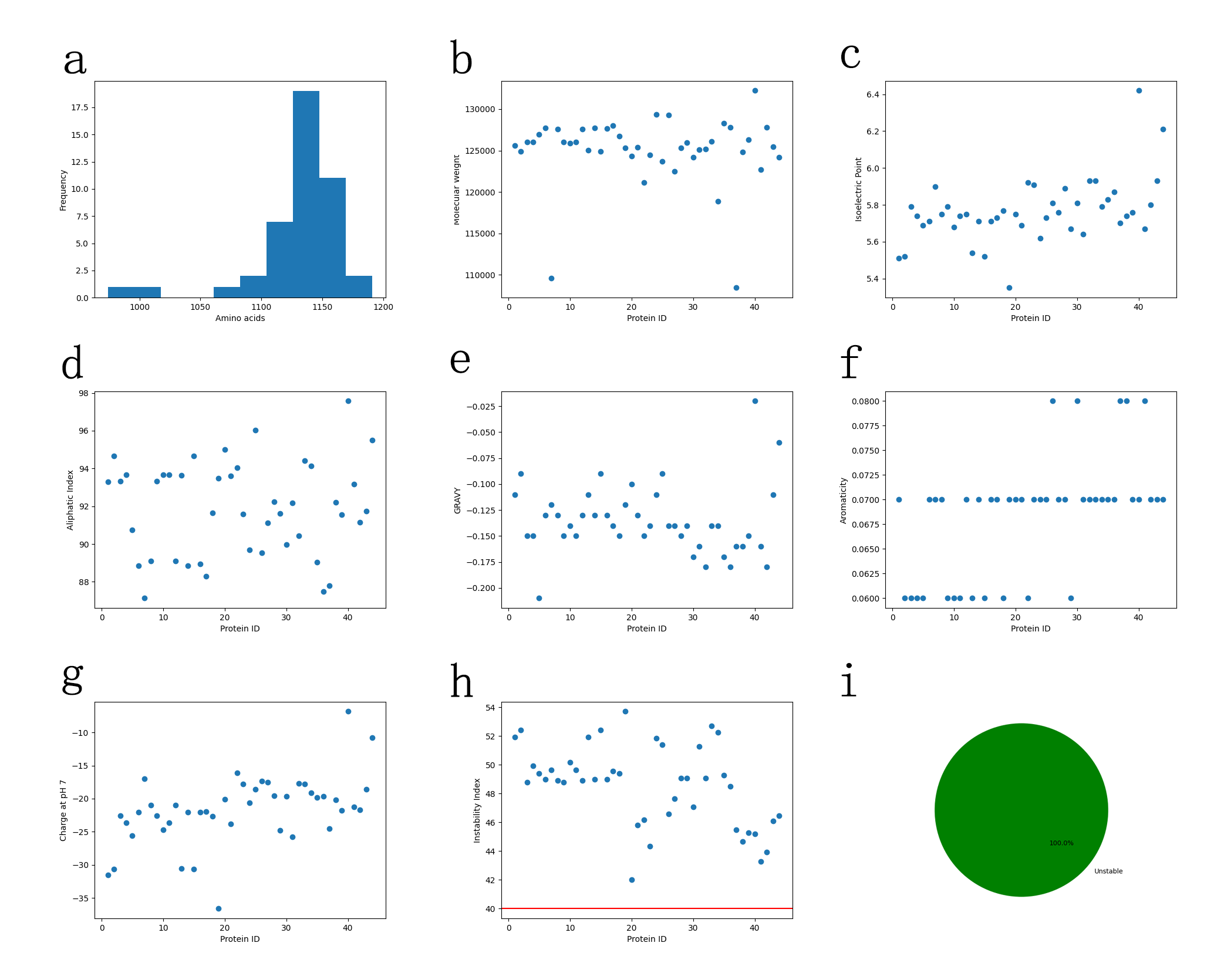

Supplement: Supplementary Figure 2 — Physicochemical properties of 44 PHYs proteins. These include amino acid length, molecular weight, theoretical isoelectric point, aliphatic index, hydropathicity (GRAVY), aromaticity, charge of pH 7, instability index and stability. [file Image2.tif]

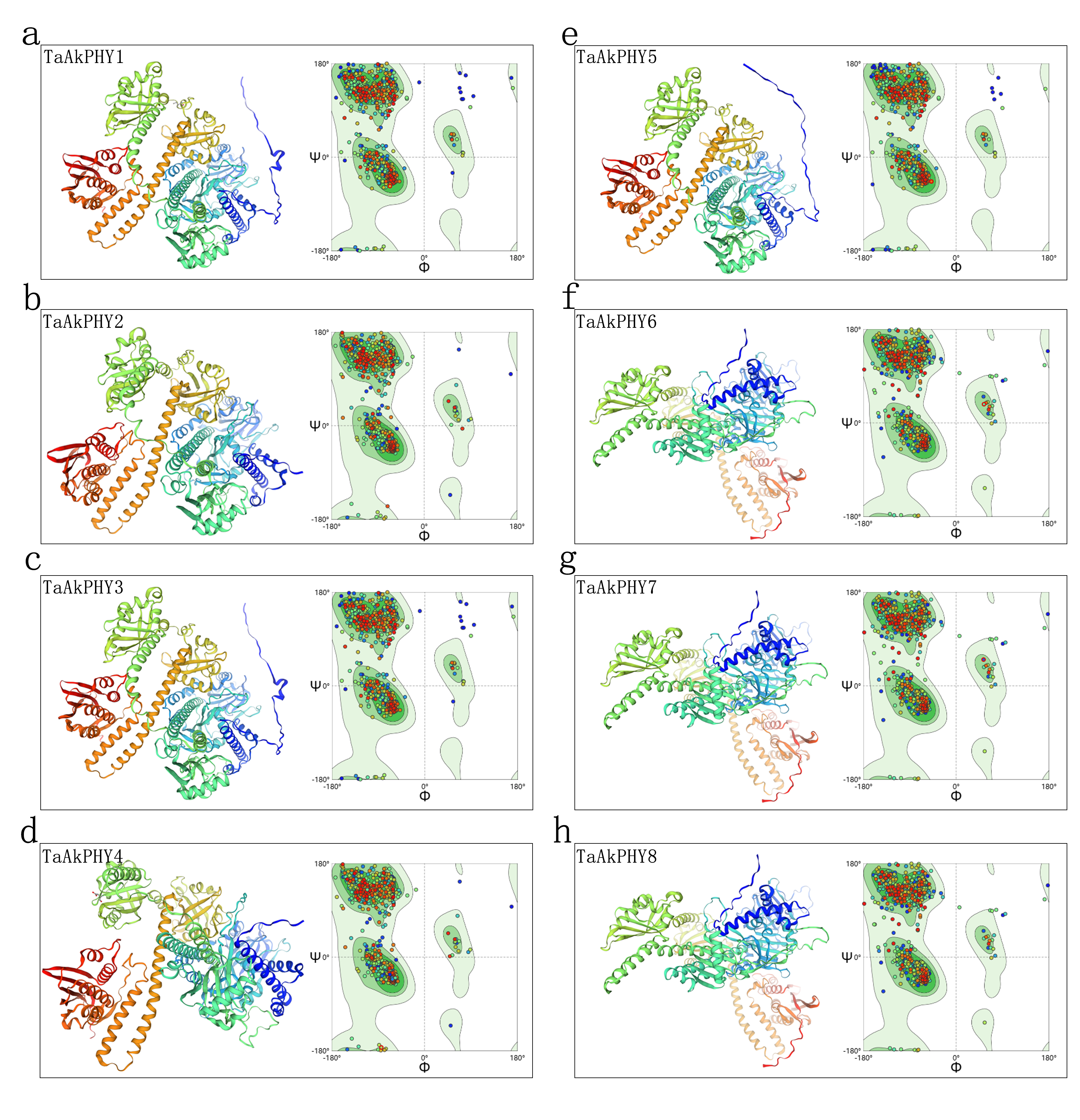

Supplement: Supplementary Figure 3 — 3D structural model and Ramachandran plot of the TaAkPHY proteins. [file Image3.tif]

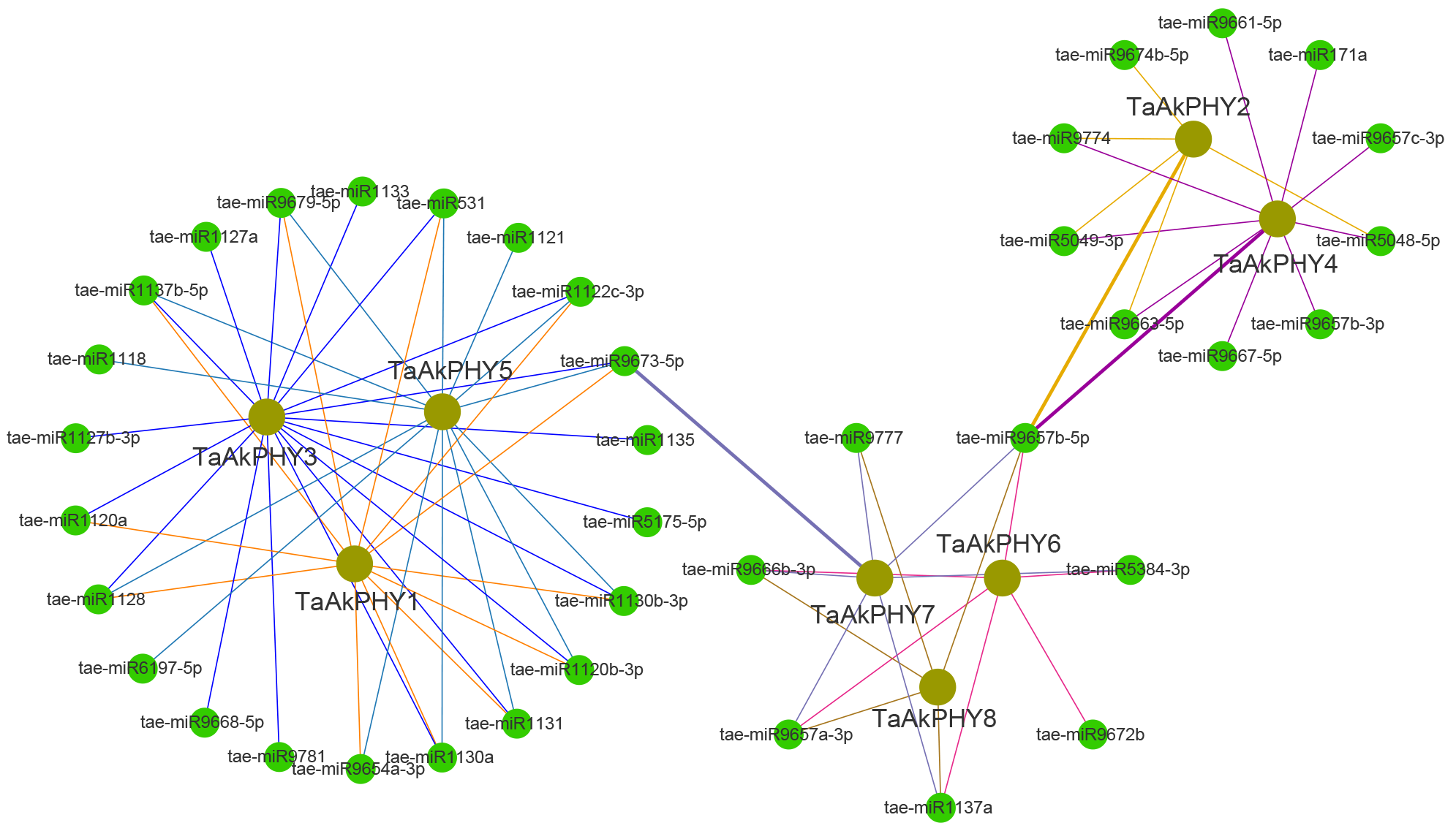

Supplement: Supplementary Figure 4 — Interaction network of targeted miRNAs and TaAkPHYs. [file Image4.tif]

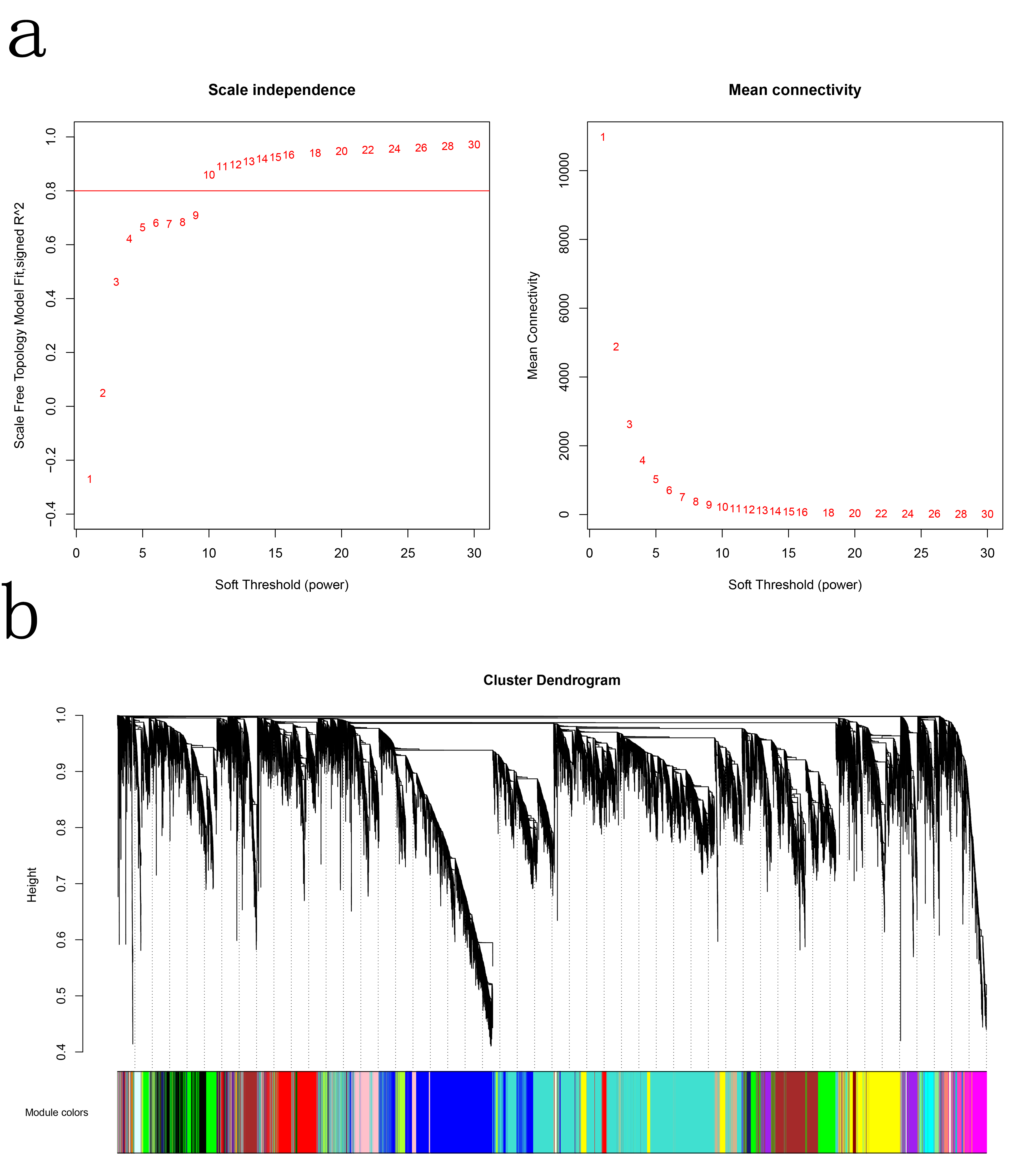

Supplement: Supplementary Figure 5 — (A) Analysis of network topology for soft threshold power. (B) Hierarchical cluster tree showing co-expression modules identified by weighted gene correlation network analysis (WGCNA) [file Image5.tif]

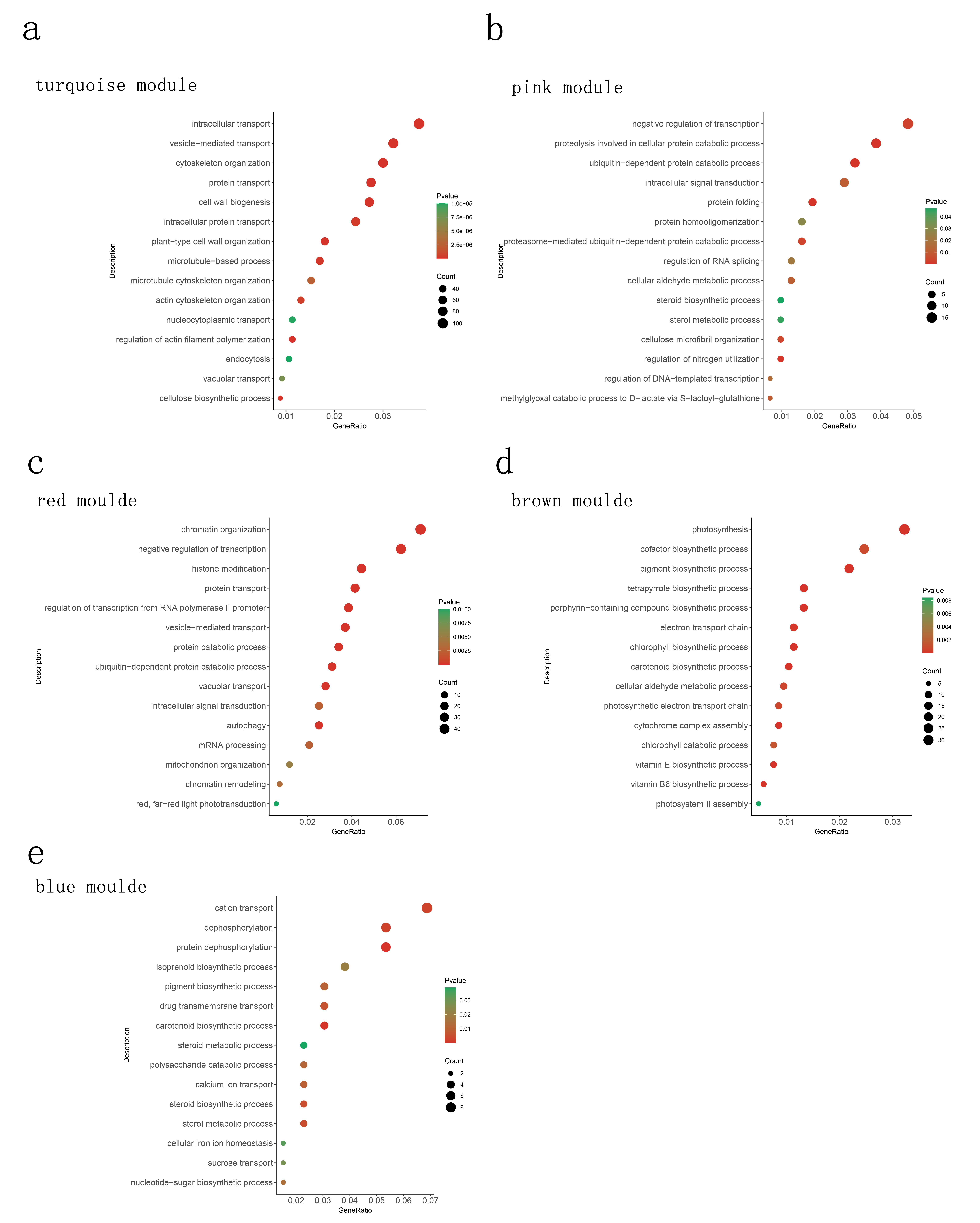

Supplement: Supplementary Figure 6 — Biological processes of GO enrichment for genes in the turquoise (B), pink (C), red (D), brown (E), and blue (F) modules. [file Image6.tif]

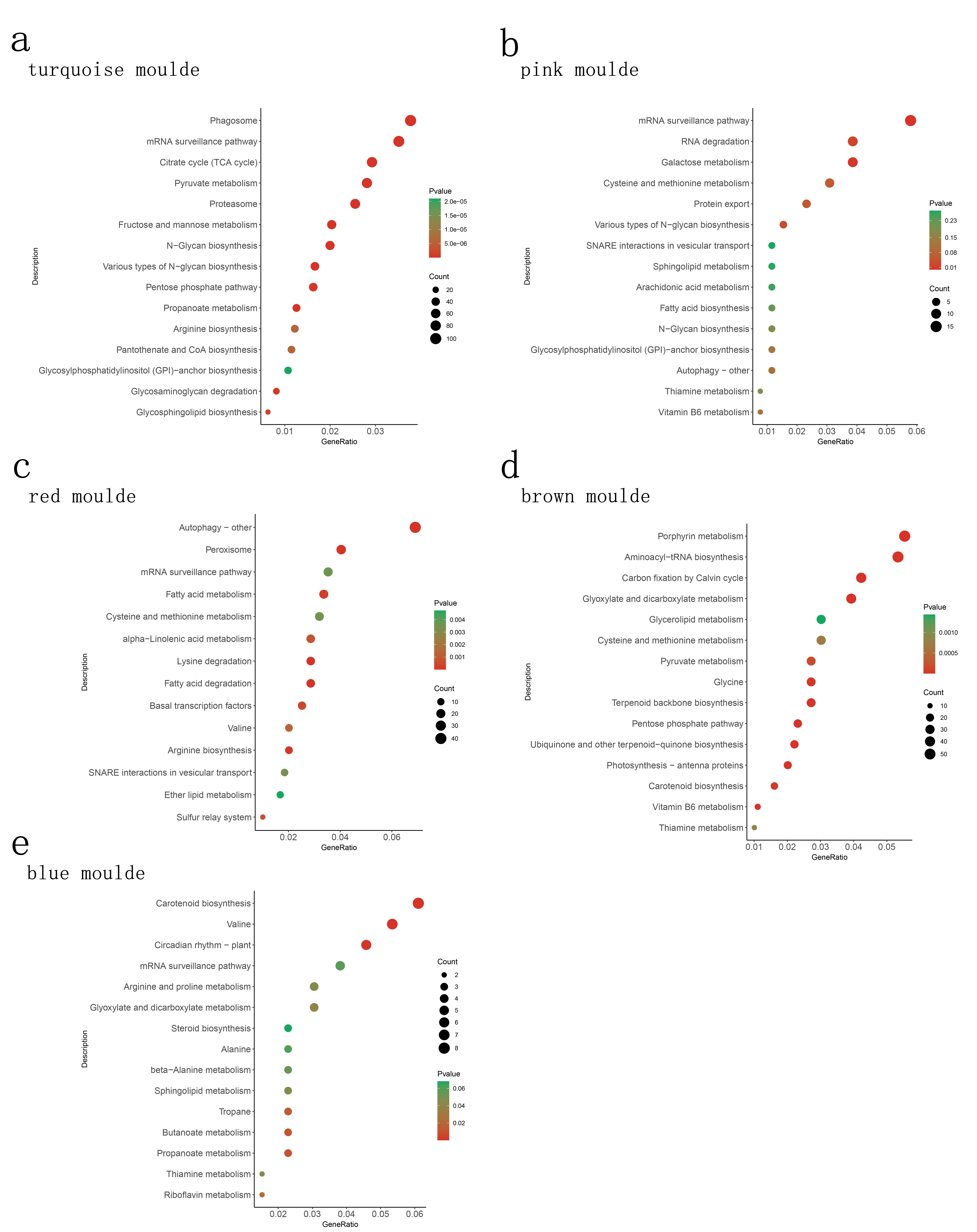

Supplement: Supplementary Figure 7 — KEGG pathway enrichment of genes in the turquoise (B), pink (C), red (D), brown (E), and blue (F) modules. [file Image7.tif]
